# Artificial intelligence based glaucoma and diabetic retinopathy detection using MATLAB — retrained AlexNet convolutional neural network

**DOI:** 10.12688/f1000research.122288.1

**Published:** 2023-01-05

**Authors:** Isaac Arias-Serrano, Paolo A. Velásquez-López, Laura N. Avila-Briones, Fanny C. Laurido-Mora, Fernando Villalba-Meneses, Andrés Tirado-Espin, Jonathan Cruz-Varela, Diego Almeida-Galárraga

**Affiliations:** 1 School of Biological Sciences and Engineering, Universidad Yachay Tech, Urcuquí, Imbabura, 100119, Ecuador; 2 Department of Design and Manufacturing Engineering, University of Zaragoza, Zaragoza, Aragon, 50018, Spain; 3 School of Mathematical and Computational Sciences, Universidad Yachay Tech, Urcuquí, Imbabura, 100119, Ecuador

**Keywords:** Glaucoma, Classification, AlexNet, Convolutional Neural Network (CNN), Diabetic Retinopathy

## Abstract

**Background:** Glaucoma and diabetic retinopathy are the leading causes of blindness due to an irreversible damage to the retina which results in vision loss. Early detection of these diseases through regular screening is especially important to prevent progression. The image of retinal fundus is the main evaluating strategy for the glaucoma and diabetic retinopathy detection. Then, automated eye disease detection is an important application of retinal image analysis. Compared with classical diagnostic techniques, image classification by convolutional neural networks (CNN) have the potential for better cost-effective performance.

**Methods:** In this paper, we propose the use of MATLAB – retrained AlexNet CNN for computerized eye diseases identification, particularly glaucoma and diabetic retinopathy, by employing retinal fundus images. The acquisition of the database was carried out through free access databases and access upon request. A transfer learning technique is used for retraining the AlexNet CNN. Specifically, the model divides the fundus image dataset into training and testing data.

**Results:** As datasets were added by network training, different values were reported for validation accuracy, false positives and false negatives, precision, and recall. Thus, having NetTransfer I with a validation accuracy value of 94.3% for two classes. NetTransfer II with a validation accuracy value of 91.8% for two classes. NetTransfer III with a validation accuracy value of 89.7% for three classes. Net transfer IV with a validation accuracy value of 93.1% for three classes. Finally, NetTransfer V with a validation accuracy value of 92.1% for three classes.

**Conclusions:** Re-training of the AlexNet network proved to be a powerful tool to create disease detection systems having high accuracy values and being able to discern between more than two diseases.

## Introduction

The leading causes of blindness and poor vision around the globe are primarily age-related eye diseases such as glaucoma and diabetic retinopathy (DR). ^1^^–^^4^ Glaucoma is a condition caused by elevated intraocular pressure. ^1^ The most common are open-angle glaucoma, angle-closure glaucoma, normal-tension glaucoma, and congenital glaucoma. ^2^ On the other hand, DR is the most frequent complication of diabetes mellitus. ^3^ It occurs because the small blood vessels in the retina swell and bleed or leak fluid, causing retinal damage and vision problems. ^3^^,^^4^ DR has five stages or classes: normal, mild, moderate, severe and proliferative DR. ^4^

Ophthalmic examination is essential for the diagnosis of glaucoma and DR. The following tests are carried out by physicians in order to perform a diagnosis for glaucoma: measuring intraocular pressure (tonometry), ^5^ analyzing optic nerve damage with a dilated eye exam, checking areas of vision loss (visual field test), ^6^ measuring corneal thickness (pachymetry) ^7^ and inspecting the angle of drainage (gonioscopy). ^8^ As most of these are imaging tests of the eye, it is essential to have accurate high quality images in order to perform a correct diagnosis of the disease. Similarly, DR is usually detected by physicians through comprehensive ophthalmologic examinations with dilation: taking cross-sectional images that show the thickness of the retina where fluid may be leaking from damaged blood vessels (optical coherence tomography) ^9^ and injecting a special dye that place blood vessels with blockages and blood vessels leaking blood (fluorescein angiography). ^10^ The diagnoses of these diseases require highly qualified medical personnel, therefore, expensive in terms of time and costs.

The artificial intelligence (AI) through deep learning methods provides computers with the ability to identify patterns in large image datasets and make predictions (predictive analysis). ^11^^–^^14^ As a consequence, it is important to use AI as a tool to automatically analyze the fundus images and assist the physicians. This results in accessible, reliable, and affordable detection of glaucoma and other pathologies in general that affect vision ( Table 1).

**Table 1.** Different deep learning systems for optical pathology detection.

Purpose	Method	Database (size)	Number of classes	Performance measure				Ref.
				Accuracy (%)	Sensitivity (%)	Specificity (%)	AUC	
Diabetic retinopathy detection and classification	Fused CNN512, CNN299, and CNN (YOLOv3, EfficientNetB0)	DDR (13673), and Kaggle (3662)	5	89.00	89.00	97.30	0.97	^15^
Diabetic retinopathy detection and classification	GoogleNet	Kaggle (200)	5	88.00	75.00	52.00	-	^16^
Automated Identification of Diabetic Retinopathy	Customized deep CNN	EyePacs, MESSIDOR 2 and E-Ophtha (75137)	2	-	74.00-94.00	80.00-98.00	0.94-0.97	^17^
Diabetic retinopathy detection and classification	CNN (modified AlexNet)	Messidor (1190)	4	95.60-96.60	88.00-96.00	97.30-96.60	-	^18^
Classification of cataract fundus image	CNN (five layers)	(7851)	2 to 4	90.82-94.07	-	-	-	^19^
Cataract diagnosis and grade	CNN (ResNet-18)	(1352)	6	92.66	-	-	-	^20^
Glaucoma detection	CNN (LeNet & U-net)	RIM-ONE, DRISHTI-GS, DRIONS-DB, JSIEC, NIO and DRIVE	2	98.8	-	99	-	^21^
Glaucoma detection	CNN	CGSA (269601)	3	-	82.2	70.4	0.82	^22^

Glossary: You only look once (YOLO), Méthodes d’Évaluation de Systèmes de Segmentation et d’Indexation Dédiées à l’Ophtalmologie Rétinienne (MESSIDOR), Residual Networks (ResNet), Retinal Image Database for Optic Nerve Evaluation (RIM-ONE), Digital Retinal Images for Optic Nerve Segmentation Database (DRIONS-DB), Joint Shantou International Eye Center (JSIEC), National Institute of Ophthalmology (NIO), Digital Retinal Images for Vessel Extraction (DRIVE), Chinese Glaucoma Study Alliance (CGSA), area under the curve (AUC), reference (Ref), convolutional neuronal network (CNN).

Related to AI, convolutional neuronal networks (CNNs) are a class of deep learning method, most commonly applied to analyze visual imagery. Generally, they are made of a key set of basic layers, including the convolution layer, the sub-sampling layer, dense layers, and the soft-max layer. ^15^^–^^22^

Among the different CNNs, AlexNet by Krizhevsky *et al.* achieved a new state-of-the-art recognition accuracy against all the traditional machine learning and computer vision approaches that offer the opportunity to be retrained. ^23^ AlexNet is structured with eight main layers, which consists of five convolutional layers (with max pooling after the first, second and fifth convolutional layer) and three fully connected layers; after each layer ReLu activation is performed except for the last one where instead there is a softmax layer that serves as the classification layer of the network. ^23^^,^^24^

**Figure 1.**
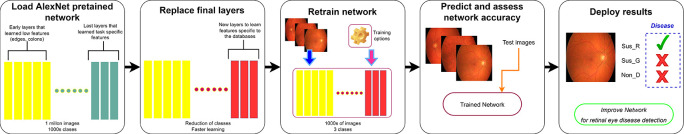
Retraining of AlexNet for non-disease (Non_D), glaucoma (Sus_G) and diabetic retinopathy (Sus_R) detection. Retinal fundus images retrieved from High-Resolution Fundus (HRF) Image Database.

Transfer learning is the process of taking a pre-trained network and use it as a starting point to learn a new task. Fine-tuning a network with transfer learning is usually much faster and easier than training a network with randomly initialized weights from scratch. Then, a pre-trained CNN quickly transfer learned features to a new task using a smaller number of training images. In this paper, we applied a transfer learning method to retrain the MatLab - AlexNet CNN for an effective glaucoma and diabetic retinopathy detection to make the testing and procedure accessible ( Figure 1).

## Methods

**Figure 2.**
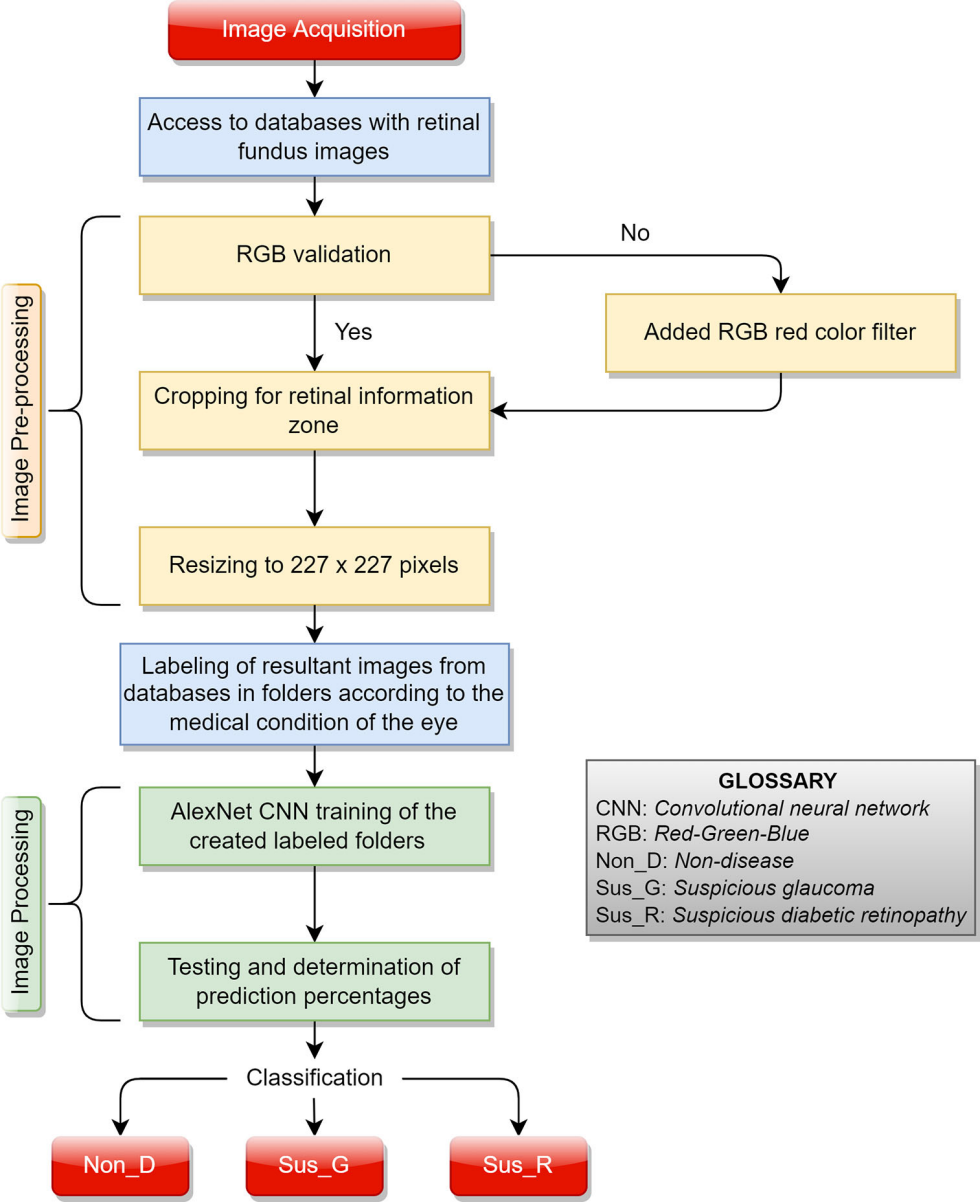
Proposed system for glaucoma and diabetic retinopathy detection using AlexNet.

To carry out the detection of glaucoma and DR through CNN, image pre-processing and processing techniques are required. The different steps are summarized in Figure 2.

### Image acquisition

For the training of the CNN it is necessary to use retinal fundus images of the eye. Several public databases that compile different eye conditions are available on the internet. In this sense, it is possible to find free access databases and databases with access upon request. The following were used in this work:

- Free access - databases
  ○ **Asia Pacific Tele-Ophthalmology Society (APTOS).** Contains 3662 images of diabetic retinopathy that were used in the APTOPS 2019 blindness screening competitions. Each image has been resized and cropped to have a maximum size of 1024px. A certified clinician rated each image according to the severity of diabetic retinopathy on a scale of 0 to 4. A directory file is provided according to the previous scale: No diabetic retinopathy (0), Mild (1), Moderate (2), Severe (3), and Proliferative diabetic retinopathy (4). ^25^
  ○ **High-Resolution Fundus (HRF) Image Database.** Contains 15 images of healthy patients, 15 images of patients with diabetic retinopathy and 15 images of glaucomatous patients. They were captured by a Canon CR-1 fundus camera with a field of view of 45 degrees with a resolution of 3504×2336px. ^26^
  ○ **Sungjoon Choi High-Resolution Fundus (sjchoi86-HRF).** Created by Sungjoon Choi, assistant professor at Korea University, contains 601 fundus images of different pixel sizes divided into 4 groups: normal (300 images), glaucoma (101 images), cataract (100 images) and retina disease (100 images). ^27^
- Access upon request – databases
  ○ **Large-scale attention based glaucoma (LAG).** Contains fundus images with positive (1711 images) and negative glaucoma (3143 images) samples obtained from Beijing Tongren Hospital with a resolution of 500×500px. Each fundus image is diagnosed by qualified glaucoma specialists, taking into consideration of both morphologic and functional analysis. ^28^
  ○ **Ocular Disease Intelligent Recognition (ODIR).** Contains images of 5000 patients with various eye diseases collected by Shanggong Medical Technology Co., Ltd. from different hospitals/medical centers in China. The fundus images are captured with various cameras on the market, resulting in varied image resolutions. They classify patients into eight labels based on the images of both eyes. A directory file is provided according to the following label: Normal Fundus (N), Diabetes (D), Glaucoma (G), Cataract (C), Age related Macular Degeneration (A), Hypertension (H), Pathological Myopia (M), Other diseases/abnormalities (O). ^29^

In the case of the ODIR database, photographs labeled in their directory file as “glaucoma” (G) and “normal fundus” (N) were extracted for a total of 200 images and 2873 images, respectively. On the other hand, for the APTOS database, photographs labeled in their directory as “moderate” (2), “severe” (3) and “proliferative diabetic retinopathy” (4) were extracted for a total of 1487 images in general.

### Image pre-processing

The AlexNet architecture design only supports color images (RGB), with a resolution of 227×227px. *Convertidor_227_final.m* code provides a small user interface that contains three functions ( *see the Software availability* links for deeper descriptions): add a red color filter to grayscale images, ^30^ crop black areas from images, and resize images of any dimension to a new 227×227px image.

The function of cropping black areas in the photograph by *Converter_227_final.m* is applied to each database. This is done to have more information on the retinal area and eliminate areas of no interest. This function binarizes the original image to obtain a black and white image of equal dimensions. Since the area where a color pixel existed now has a value of 1 and the black areas have a value of 0, the pixel location index by row and column where the value is equal to 1 is extracted as a list. Using the value of the pixel location index as image coordinates, the maximum and minimum value per row and column is determined to establish the cropping edges of the image. It should be mentioned that due to its code design, this function does not affect previously cropped images that no longer contain black areas.

Once the black borders are removed, the function to change the size of the photographs by *Converter_227_final.m* is applied. Then, all the database images are converted in a single dimension of 227×227px. Since, all databases satisfy the color image format (RGB). It was not necessary to add the red color filter by *Converter_227_final.m* code. According to their original medical classification, the obtained retinal fundus images were labeled as non-disease (Non_D), suspicious glaucoma (Sus_G) and suspicious diabetic retinopathy (Sus_R). Five storage folders were prepared for the retraining of the CNN ( Table 2).

**Table 2.** Quantity of pre-processed images used from each database for the storage folders.

Storage Folder	Database										Total images
	LAG		Sjchoi86-HRF		HRF			APTOS	ODIR		
	Non_D	Sus_G	Non_D	Sus_G	Non_D	Sus_G	Sus_R	Sus_R	Non_D	Sus_G	
1	3143	1711	-	-	-	-	-	-	-	-	4854
2			300	101							5255
3					15	15	15				5300
4								1487			6787
5									2873	200	9860

Glossary: Large-scale attention based glaucoma (LAG), Sungjoon Choi High-Resolution Fundus (sjchoi86-HRF), High-Resolution Fundus (HRF), Asia Pacific Tele-Ophthalmology Society (APTOS), Ocular Disease Intelligent Recognition (ODIR), Non-disease (Non_D), suspicious glaucoma (Sus_G) and suspicious diabetic retinopathy (Sus_R).

### Image processing

In order to develop predictive software for glaucoma disease, we are going to make use of transfer learning in order to retrain CNN AlexNet. For this, we load the pre-trained AlexNet network and also the different databases (LAG, APTOS, HFR, ODIR, and sjchoi86-HRF) containing the images of the different pathologies to be classified, thus being the glaucoma and retinopathy, besides that we use the information from Refs. 31– 38 to develop our algorithm.

In order to begin training with this new dataset, the new images should be unzipped and loaded as *ImageData* format. AlexNet will automatically label the images based on folder names, corresponding to non-disease fundus eyes images, glaucoma fundus eyes images, and retinopathy fundus eyes images. Then, the model stores the data as an *ImageData* object, we must divide the data into training and validation data sets. Once the first classification is obtained, the model further divides the data into training images having an equivalent of 70% of the images and the other 30% for validation, for this we use “. splitEachLabel” which splits the images data store into two new data stores. ^38^

***Training algorithm for transfer learning***

*Input ->Retinal fundus images (X, Y); Y = {y {Non-disease, Suspicious-Glaucoma, Suspicious-Diabetic-Retinopathy}*

*Output-> Re-trained model that classifies the retinal fundus images into respective Y*

------------------------------------------------------------------------------------------------------------------

*Import the pre-trained model AlexNet Network with its corresponding weights.*

*Replace the last three layers of the Network:*

*-Fully connected layer (Set the 'WeightLearnRateFactor' to 20 and the 'BiasLearnRateFactor' to 20; and set its output to the number of elements of Y).*

*-Softmax layer*

*-Classification layer*

***Training-progress settings***

*MinibatchSize->It is the number of elements into the group of inputs for each iteration*

*MaxEpoch->It is the maximum number of times that the network is going to use all the input elements*

*InitialLearnRate ->The learning rate is a tuning parameter that determines the step size at each iteration while moving toward a minimum of a loss function.*

*Shuffle->It is the action of mixing randomly various elements from our databases*

*ValidationData ->It is a group of images from the dataset that the network is using to Validate how good the network is getting at classification*

*ValidationFrequency ->It is the number of iterations that the system does before doing a validation process to assess in real time how the training is going*

*Verbose->Verbose mode is an option that provides additional details as to what the computer is doing and what drivers and software it is loading during startup*

***Training Code***

*options = trainingOptions('sgdm', …*

*'MiniBatchSize',10, …*

*'MaxEpochs',6, …*

*'InitialLearnRate',1e-4, …*

*'Shuffle','every-epoch', …*

*'ValidationData',augimdsValidation, …*

*'ValidationFrequency',3, …*

*'Verbose',false, …*

*'Plots','training-progress');*

**Figure 3.**
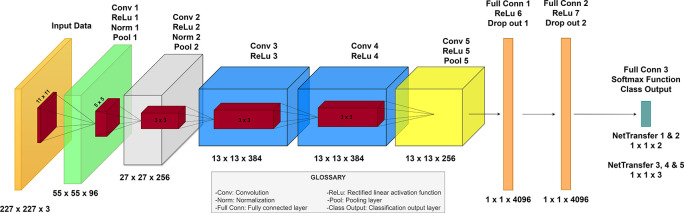
Proposed neural network architecture for eye diseases detection based on AlexNet.

The aforementioned process was applied several times with the different databases acquired. Therefore, we were able to create five new networks based on the structure and weights of the original Alexnet network, the five new networks were denominated: netTransfer 1 (Storage folder 1), netTransfer 2 (Storage folder 2), netTransfer 3 (Storage folder 3), netTranfer 4 (Storage folder 4), and netTransfer 5 (Storage folder 5). The following figure resumes the architecture of all the new networks formed during the transfer learning method ( Figure 3).

## Results

**Figure 4.**
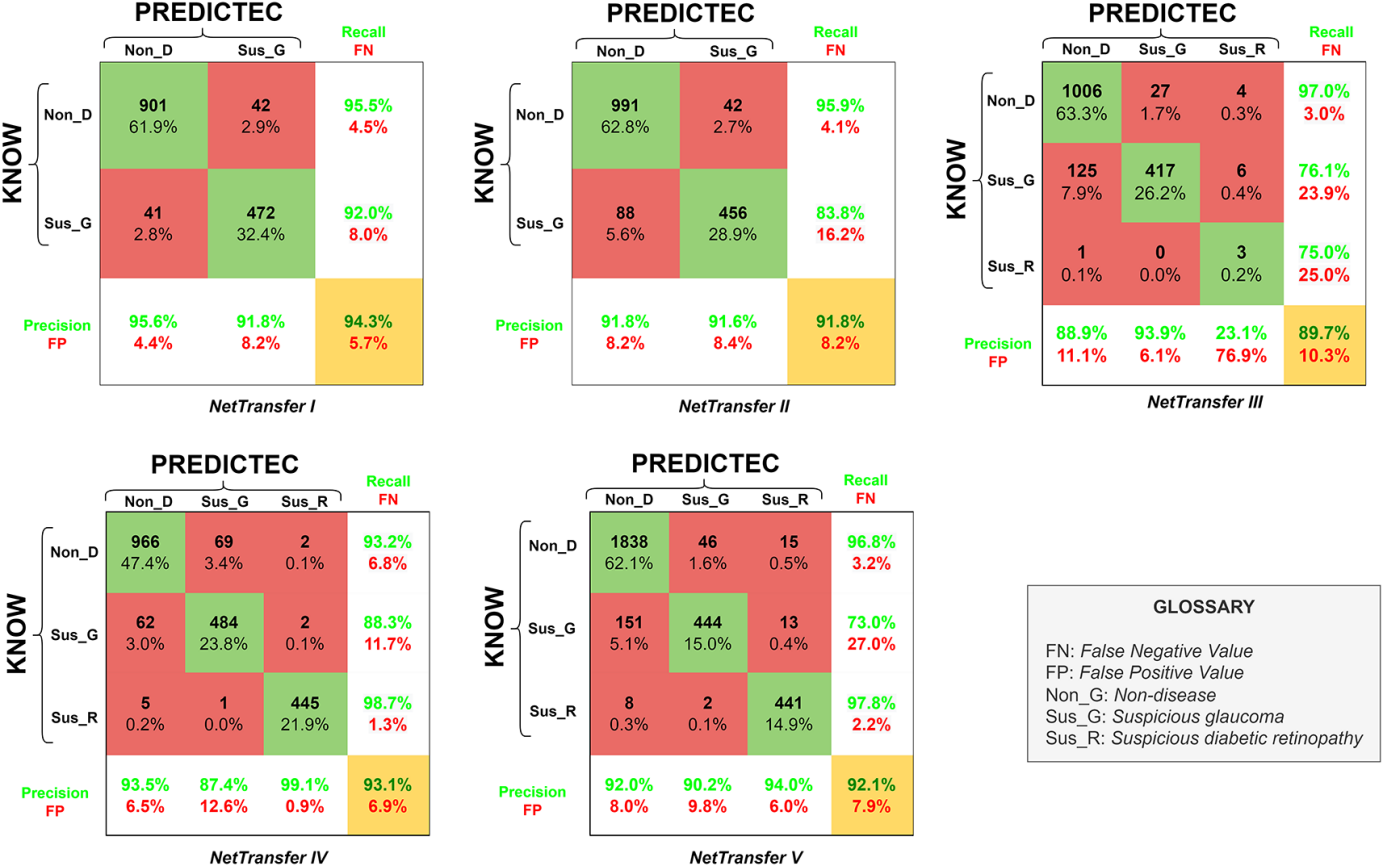
Confusion matrix for accuracy of the retrained AlexNet convolutional neural network on all datasets for the eye disease detection.

The results we obtained with our transfer learning code and our different databases over the course of the project was the development of five newly retrained AlexNet networks, to which we will now refer as NetTransfer networks. The confusion matrixes (CM) for the respective NetTransfer networks can be seen in Figure 4 including precision, recall, false positive (FP), false negative (FN) and accuracy values (yellow box). Additionally, the rows represent the known values and columns the predicted values.

NetTransfer I network was only based on glaucoma and non-disease image cases existing in the LAG-database ( Table 2), training with these datasets lead to values of validation accuracy of 94.3%. Besides that, Non_D detection also presented values of 95.5% for recall (4.5% for FN), and values of 95.6% for the precision of the system (4.4% for FP).

NetTransfer II network was based on glaucoma and non-disease images cases existing in the LAG-database and the sjchoi86-HRF database ( Table 2), training with these datasets lead to values of validation accuracy of 91.8%. Besides that, Non_D detection presented values of 95.9% for recall (4.1% for FN), and values of 91.8% for the precision of the system (8.2% for FP).

NetTransfer III network was based on glaucoma, diabetic retinopathy and non-disease images cases existing in the LAG-database, sjchoi86-HRF database and the HRF database ( Table 2), training with these datasets lead to values of validation accuracy of 89.7%. Besides that, Non_D detection presented values of 97.0% for recall (3.0% for FN), and values of 88.9% for the precision of the system (11.1% for FP).

NetTarnsfer IV network was based on glaucoma, diabetic retinopathy and non-disease images cases existing in the LAG-database, sjchoi86-HRF database, HRF database and the APTOS database ( Table 2), training with these datasets lead to values of validation accuracy of 93.1%. Besides that, Non_D detection presented values of 93.2% for recall (6.8% for FN), and values of 93.5% for the precision of the system (6.5% for FP).

NetTransfer V network was based on glaucoma, diabetic retinopathy and non-disease images cases existing in the LAG-database, sjchoi86-HRF database, HRF database, APTOS database and ODIR database ( Table 2), training with these datasets lead to values of validation accuracy of 92.1%. Besides that, Non_D detection presented values of 96.8% for recall (3.2% for FN), and values of 92.0% for the precision of the system (8.0% for FP).

## Discussion

Several works were presented for glaucoma detection using fundus photographs by calculating cup disk ratio (CDR). For example, Carrillo and coworkers ^39^ developed an autonomic detection method and a novel method for cup segmentation with a percentage of success of 88.5% ( Table 3). Another work from Anum Abdul and peers, ^40^ an algorithm was provided to detect CDR and hybrid textural and intensity features. Those features were used to classify the autonomous system, and it gave improvements in the results from previous studies that only used CDR, thanks to their hybrid approach, they reached an accuracy of 92% ( Table 3). Even though we did not make use of CDR characteristic, our AlexNet approach seems to reach comparable levels of accuracy (92.1%) to these previously mentioned methods without the need to calculate the CDR.

**Table 3.** Comparison between related studies.

Detectable pathology	Detection method	Dataset (Size)	Type of channels	Number of classes	Performance measure				Ref.
					Accuracy (%)	Sensitivity (%)	Specificity (%)	AUC	
Glaucoma and diabetic retinopathy **( *Ours*)**	CNN	LAG, APTOS, HRF, and ODIR (9860)	RGB	3	92.06	73.0 (Glaucoma) 97.80 (Retinopathy)	97.93 (Glaucoma) 98.78 (Retinopathy)	-	-
Glaucoma	Algorithm to improve glaucoma detection using cup segmentation	Set of fundus images from the CPAG in Bucaramanga, Colombia	Gray scale, each RGB color channel independently	2	88.50	-	-	-	^39^
Glaucoma	Algorithm to detect glaucoma using a fusion of CDR and hybrid textural and intensity features	Local database of 50 fundus images with 15 glaucoma and 35 healthy images	Binary image, Green and RGB	2	92.00	100.00	88.00	-	^40^
Glaucoma	DCNN	ORIGA (650) and SCES (1676)	RGB	2	-	-	-	0.83 (ORIGA) and 0.88 (SCES)	^41^
Glaucoma	CNN	CGSA (241032)	Gray scale and RGB	3	-	82.20	70.40	0.82	^22^
Glaucoma	Direct Feed Neural Network	ACRIMA (705)	Green, gray scale and binary image	2	94.61	94.57	95.00	-	^42^
Diabetic retinopathy	Customized DCNN	EyePacs, MESSIDOR 2 and E-Ophtha (for testing) (75137)	RGB	2	-	74.00-94.00	80.00-98.00	0.94-0.97	^17^
Diabetic retinopathy	CNN (modified AlexNet)	Messidor (1190)	Green and RGB	4	95.60-96.60	88.00-96.00	97.30-96.60	-	^18^
Diabetic retinopathy	GoogleNet	Kaggle (200)	RGB	5	88.00	75.00	52.00	-	^16^
Diabetic retinopathy	Fused CNN512, CNN299, and CNN (YOLOv3, EfficientNetB0)	DDR (13673), and Kaggle (3662)	RGB	5	89.00	89.00	97.30	0.97	^15^

Glossary: Large-scale attention based glaucoma (LAG), Ocular Disease Intelligent Recognition (ODIR) High-Resolution Fundus (HRF), Asia Pacific Tele-Ophthalmology Society (APTOS), Singapore Chinese Eye Study (SCES), Online Retinal Fundus Image Dataset for Glaucoma Analysis and Research (ORIGA), Center of Prevention and Attention of Glaucoma (CPAG), Cup to Disc Ratio (CDR), Red blue green (RGB), You only look once (YOLO), Méthodes d’Évaluation de Systèmes de Segmentation et d’Indexation Dédiées à l’Ophtalmologie Rétinienne (MESSIDOR), General-Purpose Database for Diabetic retinopathy (DDR), Residual Networks (ResNet), Retinal Image Database for Optic Nerve Evaluation (RIM-ONE), Digital Retinal Images for Optic Nerve Segmentation Database (DRIONS-DB), Joint Shantou International Eye Center (JSIEC), National Institute of Ophthalmology (NIO), Digital Retinal Images for Vessel Extraction (DRIVE), Chinese Glaucoma Study Alliance (CGSA), Area under the curve (AUC), reference (Ref), convolutional neuronal network (CNN), Deep convolutional neural network (DCNN).

In other more rigorous studies such as Xiangyu Chen work, ^41^ a deep CNN was developed with a total of six layers: two fully connected layers and four convolutional layers. The results drop scores of prediction from 71% to 83% from real images ( Table 3). On the other hand, Hanruo Liu and peers ^22^ made a deep learning system using a total of 241,032 images from 68,013 patients. In this work, every image was subjected to a multiple layers of grading system, in which graders were from students to senior specialists on glaucoma, from these they obtained good levels of sensitivity and specificity (82.20% and 70.40%). When compared to these other CNN systems, our AlexNet based detection system still presented comparable and even arguably better results in accuracy, sensitivity and specificity for glaucoma and DR detection ( Table 3).

Another related work from Almeida and peers, ^42^ uses image processing in MATLAB to improve the accuracy of glaucoma tests by extracting the most pertinent qualities of the images obtaining promising results with an accuracy, specificity, and sensitivity greater than 90% ( Table 3), which indicates that it gives an excellent start for us to assess the glaucoma diagnosis through AI. While Almeida and co-workers seems to be better suited for glaucoma detection, our AlexNet system presented the advantage of also being able to detect more pathologies at the same time, such as DR, which was the other disease we decided to add to our detection system.

As our system is also able to detect DR, it is also pertinent to compare it to other deep learning systems that were developed for DR detection. In the study realized by Rishab Gargeya and Theodore Leng, ^17^ they developed and evaluated a data-driven deep learning algorithm as a novel diagnostic tool for automated DR detection, which proved to reach high efficacy computer-aided model, with low-cost, which lead to correct DR diagnostics without depending on clinicians to examine and grade images manually ( Table 3). A different study made by Shanthi and Sabeenian, ^18^ used a modified AlexNet CNN system for the detection of DR in a big data training of the network ( Table 3). Additionally, Amnia Salma and peers ^16^ develop a similar system, but the use GoogleNet instead of AlexNet ( Table 3). While all of these systems follow similar principles to our propose system, it is important to remark that our AlexNet pretrained network acquired higher accuracies, sensitivities and specificities than the previously mentioned systems, mostly due to using a higher number datasets. Also we decided to try to go beyond and used AlexNet to classify more diseases at the same time. The following table summarizes and compares the capabilities of detection of our AlexNet pathology detection system to all the previously mentioned works, as well as some other important studies that were not addressed ( Table 3).

Additionally, for the implementation of the AlexNet architecture on open-source language, we endorse the use of TensorFlow which is a free open-source self-learning platform based on the Python language, mainly developed by Google. ^43^ Among its many libraries we can find Keras, which is a deep learning application programming interface (API) developed for Python built on TensorFlow, from which we can build our AlexNet equivalent model. The recommended model is the sequential model of Keras which allows a user to define the model as a series of convolutional layers with max pooling. ^44^

## Conclusions

In the presented research, the training of a CNN through the use of MATLAB software and its AlexNet tool, allowed the effective recognition of two eye diseases (glaucoma and DR) through retinal fundus images. Additionally, the use of open access databases allows the replicability and reproducibility of the present study. Being the APTOS, HRF and sjchoi86-HRF databases of immediate access. Meanwhile, LAG and ODIR are databases with access upon request. The implementation of the different databases (LAG, APTOS, HRF, ODIR, sjchoi86-HRF), proved to be effective in improving the prediction percentages of the different neural network trainings.

In general, the most common eye affections are presented through a series of symptoms, such as blurred vision, spots, glare, eye fatigue, dry eyes, among others. In this way, glaucoma proves to be a condition that damages the optic nerve and generally does not present any symptoms, until the person suffering from it perceives a decrease in vision in the final stages of the disease. Based on the foregoing, it is necessary to create tools that allow an effective detection of this type of affectation, for example CNN systems as an alternative, highly reliable in the automation of processes. Additionally, this research moved its detection objective to the addition of a new disease, such as DR.

Future improvements to this algorithm could include the creation of a more user-friendly graphical interface for users who are not experts in programming language. In this way, the detection tasks will be based on the selection of options and not on the coding of algorithms. On the other hand, as previously mentioned, it is possible to replicate the AlexNet-CNN using Python, by using existing tools such as TensorFlow and Keras API. Therefore, a subsequent study will concentrate efforts on implementing the recognition system in the open-source language, to endorse the use of non-proprietary software in order to increase reproducibility.

## Software availability

MatLab codes and scripts related to image processing, pre-processing & Training versions of the AlexNet Convolutional Neural Network ( *NetTransfers I-V*).

Source code available from: https://github.com/IscArias/EyeEvaluationSourceCode

Archived source code as at time of publication: https://doi.org/10.5281/zenodo.7098879 ^45^

License: 2-Clause BSD License

## Data availability

Free access - databases:

- Asia Pacific Tele-Ophthalmology Society (APTOS). The data download process is free and does not require authorization or registration. Follow the directions of the data host to reference the database. Link: https://www.kaggle.com/c7934597/resized-2015-2019-diabetic-retinopathy-detection/metadata/
- High-Resolution Fundus (HRF) Image Database. The data download process is free and does not require authorization or registration. Follow the directions of the data host to reference the database. Link: https://www5.cs.fau.de/research/data/fundus-images/
- Sungjoon Choi High-Resolution Fundus (sjchoi86-HRF). The data download process is free and does not require authorization or registration. Follow the directions of the data host to reference the database. Link: https://github.com/cvblab/retina_dataset

Access upon request – databases:

- Large-scale attention based glaucoma (LAG). Database for academic purposes. An email must be written to the hosts who will provide a password to authorize the download. Once the key is obtained and entered, the download is free. It is not necessary to register on any additional platform. Follow the directions of the data host to reference the database. Link: https://github.com/smilell/AG-CNN
- Ocular Disease Intelligent Recognition (ODIR). Database for academic purposes. It is required to create an account and register on the platform, providing data on the user's institution, department and country. Once registered, a request must be submitted in order to download the database. Authorized access, the user can download the database freely. Follow the directions of the data host to reference the database. Link: https://odir2019.grand-challenge.org/dataset/

### Extended data

Additional Codes for Image Evaluation, Image Selection from Data Bases and Confusion Matrix Plotting.

Source code available from: https://github.com/IscArias/EyeEvaluationSourceCode_Extra

Archived source code as at time of publication: https://doi.org/10.5281/zenodo.7102618 ^46^

License: 2-Clause BSD License
