# Artificial intelligence based glaucoma and diabetic retinopathy detection using MATLAB — retrained AlexNet convolutional neural network

**DOI:** 10.12688/f1000research.122288.2

**Published:** 2024-04-03

**Authors:** Isaac Arias-Serrano, Paolo A. Velásquez-López, Laura N. Avila-Briones, Fanny C. Laurido-Mora, Fernando Villalba-Meneses, Andrés Tirado-Espin, Jonathan Cruz-Varela, Diego Almeida-Galárraga

**Affiliations:** 1 School of Biological Sciences and Engineering, Universidad Yachay Tech, Urcuquí, Imbabura, 100119, Ecuador; 2 Department of Design and Manufacturing Engineering, University of Zaragoza, Zaragoza, Aragon, 50018, Spain; 3 School of Mathematical and Computational Sciences, Universidad Yachay Tech, Urcuquí, Imbabura, 100119, Ecuador

**Keywords:** Glaucoma, Classification, AlexNet, Convolutional Neural Network (CNN), Diabetic Retinopathy

## Abstract

**Background:**

Glaucoma and diabetic retinopathy (DR) are the leading causes of irreversible retinal damage leading to blindness. Early detection of these diseases through regular screening is especially important to prevent progression. Retinal fundus imaging serves as the principal method for diagnosing glaucoma and DR. Consequently, automated detection of eye diseases represents a significant application of retinal image analysis. Compared with classical diagnostic techniques, image classification by convolutional neural networks (CNN) exhibits potential for effective eye disease detection.

**Methods:**

This paper proposes the use of MATLAB – retrained AlexNet CNN for computerized eye diseases identification, particularly glaucoma and diabetic retinopathy, by employing retinal fundus images. The acquisition of the database was carried out through free access databases and access upon request. A transfer learning technique was employed to retrain the AlexNet CNN for non-disease (Non_D), glaucoma (Sus_G) and diabetic retinopathy (Sus_R) classification. Moreover, model benchmarking was conducted using ResNet50 and GoogLeNet architectures. A Grad-CAM analysis is also incorporated for each eye condition examined.

**Results:**

Metrics for validation accuracy, false positives, false negatives, precision, and recall were reported. Validation accuracies for the NetTransfer (I-V) and netAlexNet ranged from 89.7% to 94.3%, demonstrating varied effectiveness in identifying Non_D, Sus_G, and Sus_R categories, with netAlexNet achieving a 93.2% accuracy in the benchmarking of models against netResNet50 at 93.8% and netGoogLeNet at 90.4%.

**Conclusions:**

This study demonstrates the efficacy of using a MATLAB-retrained AlexNet CNN for detecting glaucoma and diabetic retinopathy. It emphasizes the need for automated early detection tools, proposing CNNs as accessible solutions without replacing existing technologies.

## Introduction

The leading causes of blindness and poor vision around the globe are primarily age-related eye diseases such as glaucoma and diabetic retinopathy (DR). ^1^^–^^4^ Glaucoma is a condition caused by elevated intraocular pressure. ^1^ The most common are open-angle glaucoma, angle-closure glaucoma, normal-tension glaucoma, and congenital glaucoma. ^2^ On the other hand, DR is the most frequent complication of diabetes mellitus. ^3^ It occurs because the small blood vessels in the retina swell and bleed or leak fluid, causing retinal damage and vision problems. ^3^^,^^4^ DR has five stages or classes: normal, mild, moderate, severe and proliferative DR. ^4^

Ophthalmic examination is essential for the diagnosis of glaucoma and DR. The following tests are carried out by physicians in order to perform a diagnosis for glaucoma: measuring intraocular pressure (tonometry), ^5^ analyzing optic nerve damage with a dilated eye exam, checking areas of vision loss (visual field test), ^6^ measuring corneal thickness (pachymetry) ^7^ and inspecting the angle of drainage (gonioscopy). ^8^ As most of these are imaging tests of the eye, it is essential to have accurate high quality images in order to perform a correct diagnosis of the disease.

Similarly, DR is usually detected by physicians through comprehensive ophthalmologic examinations requiring pupil dilation. This to facilitate detailed cross-sectional imaging that show the thickness of the retina where fluid may be leaking from damaged blood vessels (optical coherence tomography) ^9^ and injecting a special dye that place blood vessels with blockages plus blood vessels leaking blood (fluorescein angiography). ^10^ Diagnosing these conditions necessitates the expertise of specialized medical professionals, resulting in significant time and financial costs.

Furthermore, discrete diagnostic approaches are essential for each disease. Given the potential coexistence of diabetes with both conditions, a diagnosis of diabetes does not preclude the possibility of glaucoma. ^11^ This knowledge from medical professionals in identifying glaucoma and diabetic retinopathy is beneficial, enabling the creation of accurately labelled large databases. Such groundwork allows for the analysis of data based on established truths, facilitating the development of classification models by non-medical experts.

Artificial intelligence (AI), through deep learning methods, enables classification models with the capability to identify patterns within extensive image datasets for predictive analysis. ^12^^–^^15^ Consequently, employing AI for the automated analysis of fundus images can assist physicians by facilitating accessible, reliable, and affordable detection of glaucoma and other related visual pathologies ( Table 1).

**Table 1.** Different deep learning systems for optical pathology detection.

Purpose	Method	Database (size)	Number of classes	Performance measure				Ref.
				Accuracy (%)	Sensitivity (%)	Specificity (%)	AUC	
Diabetic retinopathy detection and classification	Fused CNN512, CNN299, and CNN (YOLOv3, EfficientNetB0)	DDR (13673), and Kaggle (3662)	5	89.00	89.00	97.30	0.97	^16^
Diabetic retinopathy detection and classification	GoogleNet	Kaggle (200)	5	88.00	75.00	52.00	-	^17^
Automated Identification of Diabetic Retinopathy	Customized deep CNN	EyePacs, MESSIDOR 2 and E-Ophtha (75137)	2	-	74.00-94.00	80.00-98.00	0.94-0.97	^18^
Diabetic retinopathy detection and classification	CNN (modified AlexNet)	Messidor (1190)	4	95.60-96.60	88.00-96.00	97.30-96.60	-	^19^
Classification of cataract fundus image	CNN (five layers)	(7851)	2 to 4	90.82-94.07	-	-	-	^20^
Cataract diagnosis and grade	CNN (ResNet-18)	(1352)	6	92.66	-	-	-	^21^
Glaucoma detection	CNN (LeNet & U-net)	RIM-ONE, DRISHTI-GS, DRIONS-DB, JSIEC, NIO and DRIVE	2	98.8	-	99	-	^22^
Glaucoma detection	CNN	CGSA (269601)	3	-	82.2	70.4	0.82	^23^

Glossary: You only look once (YOLO), Méthodes d’Évaluation de Systèmes de Segmentation et d’Indexation Dédiées à l’Ophtalmologie Rétinienne (MESSIDOR), Residual Networks (ResNet), Retinal Image Database for Optic Nerve Evaluation (RIM-ONE), Digital Retinal Images for Optic Nerve Segmentation Database (DRIONS-DB), Joint Shantou International Eye Center (JSIEC), National Institute of Ophthalmology (NIO), Digital Retinal Images for Vessel Extraction (DRIVE), Chinese Glaucoma Study Alliance (CGSA), area under the curve (AUC), reference (Ref), convolutional neuronal network (CNN).

Convolutional neuronal networks (CNNs) are a class of deep learning method, most commonly applied to analyse visual imagery. Characteristically, CNNs comprise a sequence of fundamental layers: the convolutional layer for extracting features, the pooling (sub-sampling) layer for dimensionality reduction, fully connected (dense) layers for pattern recognition, and the softmax layer for classification probabilities. ^16^^–^^23^

Among the diverse CNNs, AlexNet, by Krizhevsky *et al.* achieved a new state-of-the-art recognition accuracy against all conventional machine learning and computer vision approaches that offer the opportunity to be retrained. ^24^ AlexNet has sustained its significance as a neural network, attributed to the simplicity of its architecture, enabling its operation without necessitating substantial computational resources. As a result, AlexNet is structured with eight main layers, incorporating five convolutional layers—max pooling after the first, second and fifth convolutional layer—and three fully connected layers. Activation via the Rectified Linear Unit (ReLU) function is applied after each layer, with the exception of the final layer, which employs a softmax layer to function as the classification mechanism of the trained network. ^24^^,^^25^

**Figure 1.**
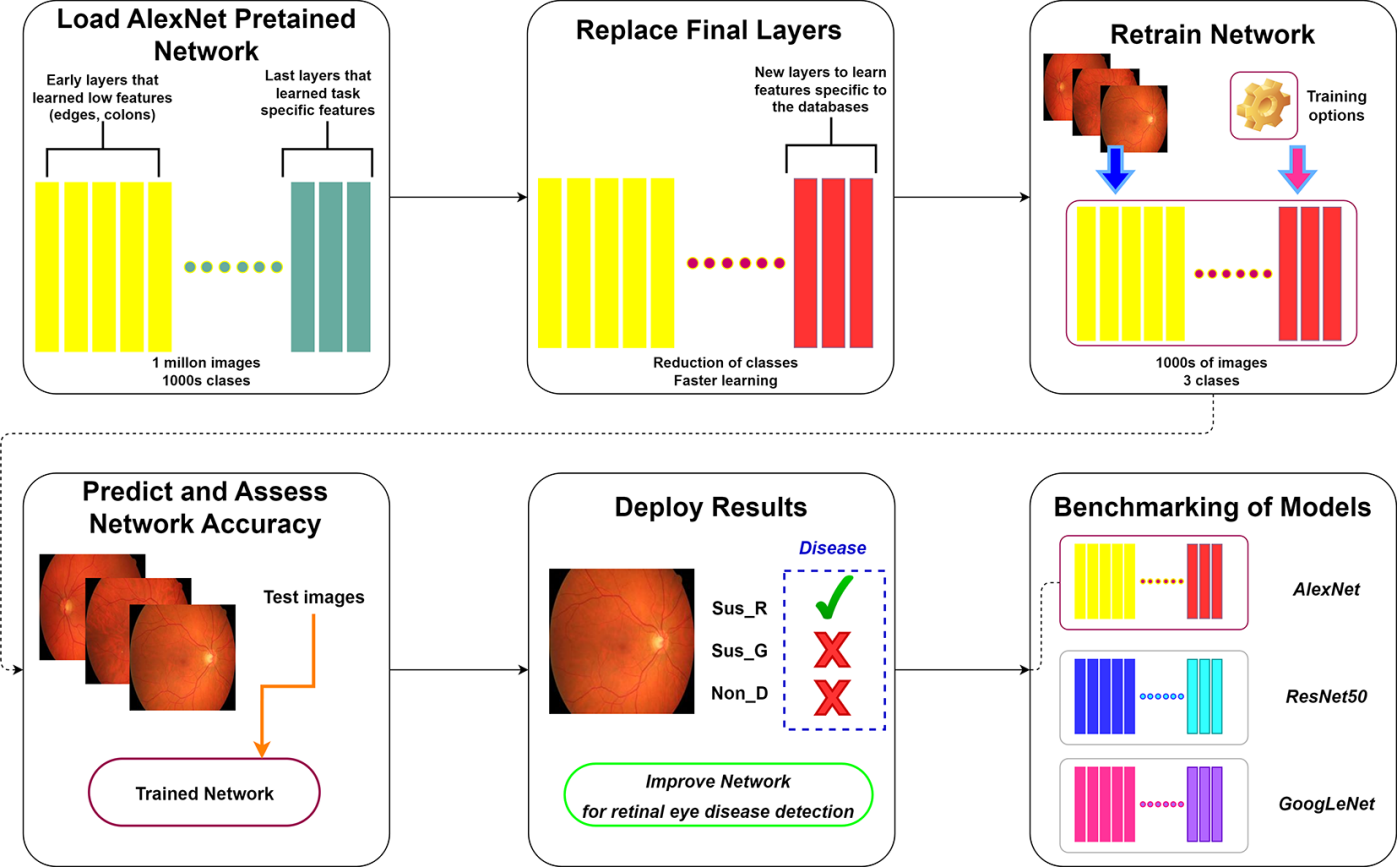
Retraining and benchmarking of AlexNet for non-disease (Non_D), glaucoma (Sus_G) and diabetic retinopathy (Sus_R) detection. Retinal fundus images retrieved from High-Resolution Fundus (HRF) Image Database.

Transfer learning involves utilizing a pre-trained network as a base model to learn a new task. This approach, notably through fine-tuning, proves being more efficient and simpler than training a network from the ground up with randomly initialized weights. As a result, the pre-trained CNN quickly transfer learned features using a smaller number of training images. In this paper, a transfer learning method to retrain the MatLab - AlexNet CNN is applied for an effective glaucoma and DR detection, aiming to make the aided recognition procedure through a low-complexity CNN accessible. Additionally, the final trained model is benchmarked against the ResNet50 ^26^ and GoogLeNet ^27^ architectures to evaluate comparative performance ( Figure 1). Furthermore, a Grad-CAM analysis across these architectures is conducted to elucidate the focal points of observation within different models.

## Methods

**Figure 2.**
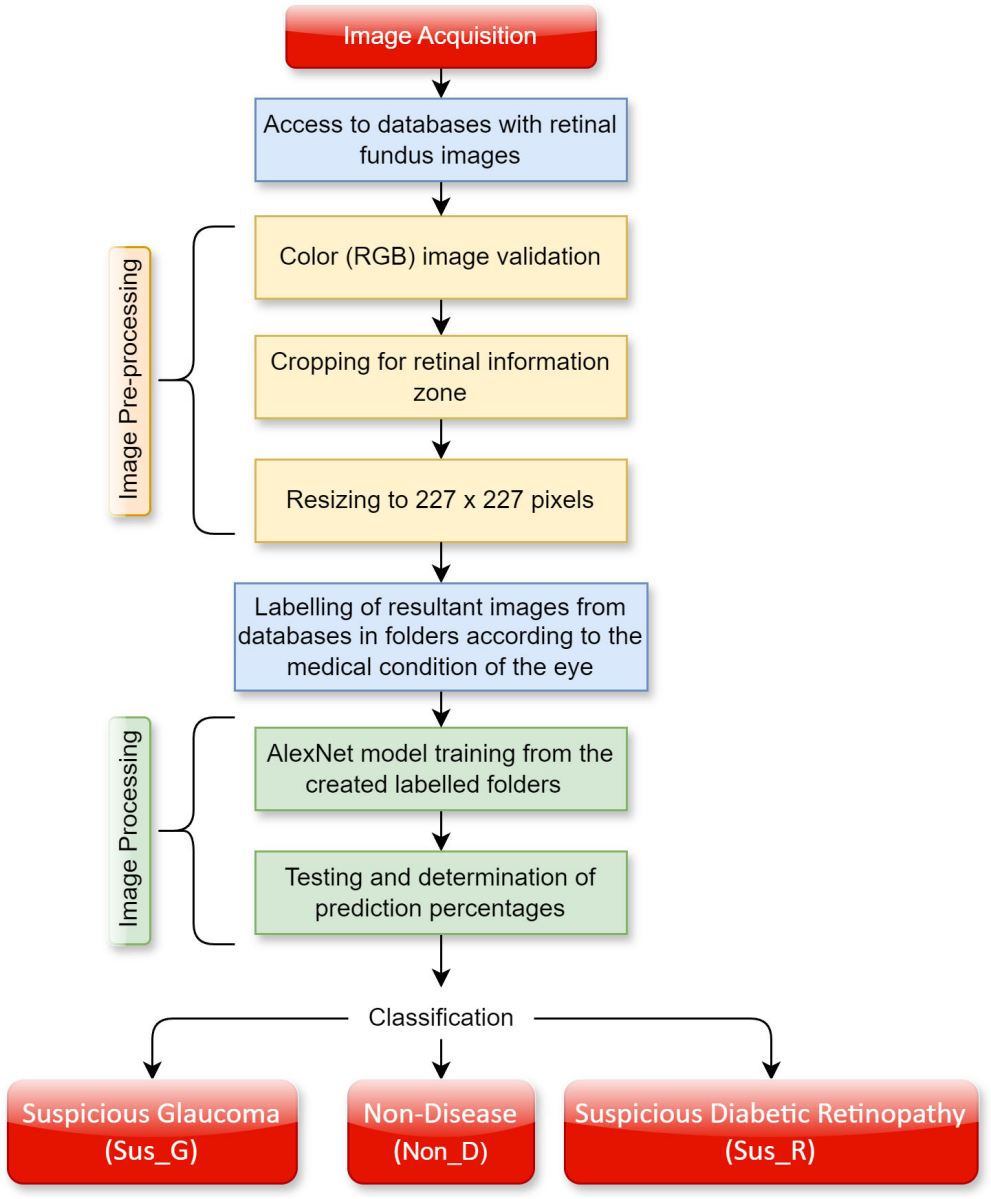
Proposed system for glaucoma and diabetic retinopathy detection using AlexNet.

To carry out the detection of glaucoma and DR through CNN, image pre-processing and processing techniques are required. The different steps are summarized in Figure 2.

### Image acquisition

For the training of the CNN it is necessary to use retinal fundus images of the eye. Several public databases that compile different eye conditions are available on the internet. In this sense, it is possible to find free access databases and databases with access upon request. The following were used in this work:

- Free access - databases
  ○ **Asia Pacific Tele-Ophthalmology Society (APTOS).** Contains 3662 images of diabetic retinopathy that were used in the APTOPS 2019 blindness screening competitions. Each image has been resized and cropped to have a maximum size of 1024px. A certified clinician rated each image according to the severity of diabetic retinopathy on a scale of 0 to 4. A directory file is provided according to the previous scale: No diabetic retinopathy (0), Mild (1), Moderate (2), Severe (3), and Proliferative diabetic retinopathy (4). ^28^
  ○ **High-Resolution Fundus (HRF) Image Database.** Contains 15 images of healthy patients, 15 images of patients with diabetic retinopathy and 15 images of glaucomatous patients. They were captured by a Canon CR-1 fundus camera with a field of view of 45 degrees with a resolution of 3504×2336px. ^29^
  ○ **Sungjoon Choi High-Resolution Fundus (sjchoi86-HRF).** Created by Sungjoon Choi, assistant professor at Korea University, contains 601 fundus images of different pixel sizes divided into 4 groups: normal (300 images), glaucoma (101 images), cataract (100 images) and retina disease (100 images). ^30^
- Access upon request – databases
  ○ **Large-scale attention based glaucoma (LAG).** Contains fundus images with positive (1711 images) and negative glaucoma (3143 images) samples obtained from Beijing Tongren Hospital with a resolution of 500×500px. Each fundus image is diagnosed by qualified glaucoma specialists, taking into consideration of both morphologic and functional analysis. ^31^
  ○ **Ocular Disease Intelligent Recognition (ODIR).** Contains images of 5000 patients with various eye diseases collected by Shanggong Medical Technology Co., Ltd. from different hospitals/medical centers in China. The fundus images are captured with various cameras on the market, resulting in varied image resolutions. They classify patients into eight labels based on the images of both eyes. A directory file is provided according to the following label: Normal Fundus (N), Diabetes (D), Glaucoma (G), Cataract (C), Age related Macular Degeneration (A), Hypertension (H), Pathological Myopia (M), Other diseases/abnormalities (O). ^32^

In the case of the ODIR database, photographs labeled in their directory file as “glaucoma” (G) and “normal fundus” (N) were extracted for a total of 200 images and 2873 images, respectively. On the other hand, for the APTOS database, photographs labeled in their directory as “moderate” (2), “severe” (3) and “proliferative diabetic retinopathy” (4) were extracted for a total of 1487 images in general.

### Operational resources

All data were processed according to the following specifications:

Software:

• MATLAB ^®^ (R2020a, By MathWorks, Natick, MA, USA)
• Deep Network Designer - MATLAB ^®^

Hardware:

• AMD Ryzen 5 4600H with Radeon Graphics (3.00 GHz)
• NVIDIA GeForce GTX 1650 GPU
• 16 GB RAM DDR4 Memory

### Image pre-processing

AlexNet architecture is specifically designed for processing color (RGB) images with a resolution of 227×227 pixels. Image pre-processing from databases is conducted using the custom function *Convertidor_227_final.m* (refer to software availability), which includes a user interface for cropping black areas and resizing images of any dimension to the required 227×227 pixel format.

The function of cropping black areas in the photograph by *Convertidor_227_final.m* is applied to each database. This is done to have more information on the retinal area and eliminate areas of no interest. This function binarizes the original image to obtain a black and white image of equal dimensions. Since the area where a color pixel existed now has a value of 1 and the black areas have a value of 0, the pixel location index by row and column where the value is equal to 1 is extracted as a list. Using the value of the pixel location index as image coordinates, the maximum and minimum value per row and column is determined to establish the cropping edges of the image. It should be mentioned that due to its code design, this function does not affect previously cropped images that no longer contain black areas.

Following the removal of black borders, the *Convertidor_227_final.m* function is employed to resize the photographs. Subsequently, all images within the database are standardized to a uniform dimension of 227×227 pixels. According to their original medical classification, the obtained retinal fundus images were labeled as non-disease (Non_D), suspicious glaucoma (Sus_G) and suspicious diabetic retinopathy (Sus_R). For the purposes of CNN re-training, five distinct storage folders were organized ( Table 2).

**Table 2.** Quantity of pre-processed images used from each database for the storage folders.

Storage Folder	Database										Total images
	LAG		Sjchoi86-HRF		HRF			APTOS	ODIR		
	Non_D	Sus_G	Non_D	Sus_G	Non_D	Sus_G	Sus_R	Sus_R	Non_D	Sus_G	
1	3143	1711	-	-	-	-	-	-	-	-	4854
2			300	101							5255
3					15	15	15				5300
4								1487			6787
5									2873	200	9860

Glossary: Large-scale attention based glaucoma (LAG), Sungjoon Choi High-Resolution Fundus (sjchoi86-HRF), High-Resolution Fundus (HRF), Asia Pacific Tele-Ophthalmology Society (APTOS), Ocular Disease Intelligent Recognition (ODIR), Non-disease (Non_D), suspicious glaucoma (Sus_G) and suspicious diabetic retinopathy (Sus_R).

### Image processing

To develop the predictive software for eye disease detection, transfer learning is utilized to retrain the CNN AlexNet. The pre-trained AlexNet network is loaded alongside the different databases (LAG, APTOS, HFR, ODIR, and sjchoi86-HRF) that containing the images of the different pathologies to be classified, specifically glaucoma and retinopathy. Information from Refs. 33– 40 is employed to develop our algorithm.

To initiate dataset training, image storage folders as outlined in Table 2 are created. Images are stored in a primary folder with corresponding subfolders Non_D, Sus_G, and Sus_R, based on the original classification assigned within their respective databases. The primary database is loaded as an “ *imds*” variable, and the data contained within the subfolders are segmented into training and validation sets. A conventional data division approach is applied, allocating 70% of the images for training and 30% for validation using the “ *splitEachLabel (imds,0.7,‘randomized’)*” function. ^40^ This method randomly splits the data in the image datastore *“imds”* into two new datastores.

**Figure 3.**
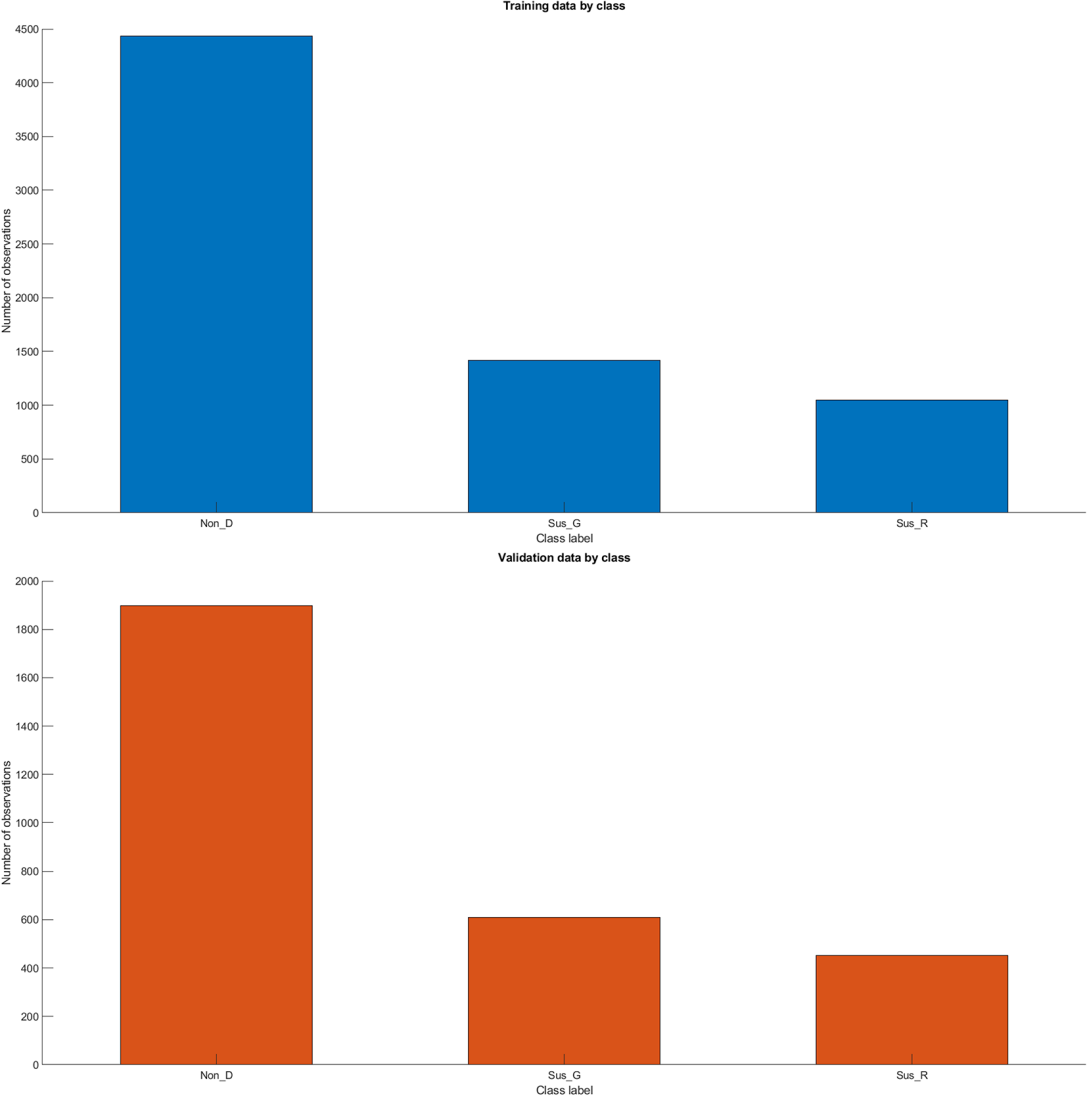
Data Organization generated by MATLAB for Training and Validation Sets. Glossary: Non-disease (Non_D), glaucoma (Sus_G) and diabetic retinopathy (Sus_R).

MATLAB allocates 70% of the images from each label (or subfolder) in *“imds”* for training and the remaining 30% for validation, with the selection done in a randomized manner. This ensures that the training and validation datasets are representative of the overall dataset, enhancing the generalizability of the model trained on this data. A representation of the data split generated by MATLAB is presented for the maximum data volume of 9,680 observations see Figure 3.

Alexnet was applied to binary classification, distinguishing retinal fundus images as Non_D vs. Sus_G (NetTransfer I & II), and multi-class classification, differentiating among Sus_G, Sus_R and Non_D categories (NetTransfer III, IV & V). Each image storage folder, as detailed in Table 2, underwent training with the corresponding NetTransfer model number. The subsequent pseudocode outlines the procedure for both binary and multi-class classification tasks in AlexNet.

***Training algorithm for transfer learning***

*Input ->Retinal fundus images (X, Y); Y = {y {Non-disease, Suspicious-Glaucoma, Suspicious-Diabetic-Retinopathy}*

*Output-> Re-trained model that classifies the retinal fundus images into respective Y*

------------------------------------------------------------------------------------------------------------------

*Import the pre-trained model AlexNet Network with its corresponding weights.*

*Replace the last three layers of the Network:*

*-Fully connected layer (Set the 'WeightLearnRateFactor' to 20 and the 'BiasLearnRateFactor' to 20; and set its output to the number of elements of Y).*

*-Softmax layer*

*-Classification layer*

***Training-progress settings***

*MinibatchSize->It is the number of elements into the group of inputs for each iteration*

*MaxEpoch->It is the maximum number of times that the network is going to use all the input elements*

*InitialLearnRate ->The learning rate is a tuning parameter that determines the step size at each iteration while moving toward a minimum of a loss function.*

*Shuffle->It is the action of mixing randomly various elements from our databases*

*ValidationData ->It is a group of images from the dataset that the network is using to Validate how good the network is getting at classification*

*ValidationFrequency ->It is the number of iterations that the system does before doing a validation process to assess in real time how the training is going*

*Verbose->Verbose mode is an option that provides additional details as to what the computer is doing and what drivers and software it is loading during startup*

***Training Code***

*options = trainingOptions('sgdm', …*

*'MiniBatchSize',10, …*

*'MaxEpochs',6, …*

*'InitialLearnRate',1e-4, …*

*'Shuffle','every-epoch', …*

*'ValidationData',augimdsValidation, …*

*'ValidationFrequency',3, …*

*'Verbose',false, …*

*'Plots','training-progress');*

The MiniBatchSize (MBS) parameter specifies the number of observations processed and used to update the weights of the model in each iteration. By setting the MBS to 10, the observations in the Training Set (TS) are divided by this number to calculate the Iterations Per Epoch (IPE). This division ensures each observation is utilized once per epoch, reducing biases in the training process. Thus, every iteration involves processing a mini-batch of data, executing a forward pass through the network, calculating the error, and adjusting the weights.

Models employed in transfer learning technique have previously undergone training on extensive and generalized datasets, such as ImageNet for CNNs, ^24^ emphasizing fine-tuning over learning from scratch. Consequently, “MaxEpochs” is set as 6 for model evaluation, given transfer learning technique typically requires “tuning” the weights of the pre-trained model to suit a new specific task rather than acquiring all features once again. This fine-tuning process demands fewer modifications to the weights, achievable within a limited number of epochs. Table 3 provides a comprehensive overview of the underlying mathematics for each model, detailing calculations related to database size and the corresponding derived training parameters.

**Table 3.** Mathematical framework for model training parameters based on database size.

Net Transfer	OBS	TS (OBS x 70%)	VS (OBS x 30%)	MBS	IPE (TS/MBS)	EPOCHS	MI (EPOCH X IPE)
I	4854	3398 ≈ (4854 x 0.7)	1456 ≈ (4854 x 0.3)	10	340 ≈ (3398/10)	6	2040 = (6 x 340)
II	5255	3678 ≈ (5255 x 0.7)	1577 ≈ (5255 x 0.3)	10	368 ≈ (3678/10)	6	2208 = (6 x 368)
III	5300	3710 ≈ (5300 x 0.7)	1590 ≈ (5300 x 0.3)	10	371 ≈ (3710/10)	6	2226 = (6 x 371)
IV	6787	4751 ≈ (6787 x 0.7)	2036 ≈ (6787 x 0.3)	10	475 ≈ (4751/10)	6	2850 = (6 x 368)
V	9860	6902 ≈ (9860 x 0.7)	2958 ≈ (9860 x 0.3)	10	690 ≈ (6902/10)	6	4140 = (6 x 690)

Glossary: Observations (OBS), Test Set (TS), Validation Set (VS), Mini Batch Size (MBS), Iteration Per Epoch (IPE), Maximum Iterations (MI).

**Figure 4.**
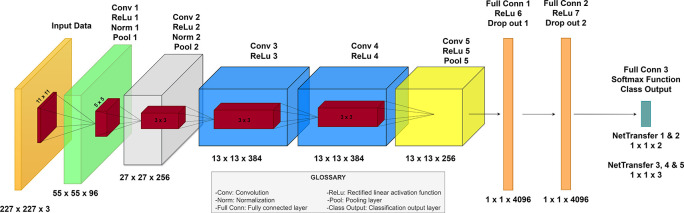
Proposed neural network architecture for eye diseases detection based on AlexNet.

Furthermore, a “ValidationFrequency” of 3 was chosen due to the relatively low epoch count of the model (six in this instance), and an “InitialLearnRate” of 0.0001 was selected as a conservative value to facilitate gradual adjustments to the weights of the model. The following figure resumes the architecture of all the new networks designed during the transfer learning technique ( Figure 4).

### Benchmarking of models

The performance comparison between the architectures of AlexNet, GoogLeNet, and ResNet50 was conducted through multi-class classification of the categories Sus_G, Sus_R, and Non_D. Given that data storage 5 represents the most complete dataset, it was chosen as the input data storage for the training of three new models (netALEXNET, netRESNET50, and netGOOGLENET). To accelerate the model design of these networks, the “deepNetworkDesigner” function was utilized, application aimed for network architecture and transfer learning techniques through a user-friendly interface (refer to software availability).

In this context, the construction and training of the models were carried out under identical conditions for data loading and handling as those used in NetTransfer I-V, with feedback provided only for the parameters MBS, EPOCHS, and ValidationFrequency. For ResNet50, the model loading exceeded our computational capabilities, necessitating a reduction in MBS to align it with the IPE (see Table 4).

**Table 4.** Computational Adjustments for AlexNet, GoogLeNet, and ResNet50 Training Parameters.

MODEL	OBS	TS (OBS x 70%)	VS (OBS x 30%)	MBS	IPE (TS/MBS)	EPOCHS	MI (EPOCH X IPE)
AlexNet	9860	6902 ≈ (9860 x 0.7)	2958 ≈ (9860 x 0.3)	138	50 ≈ (6902/138)	30	1500 = (30 x 50)
ResNet50				50	138 ≈ (6902/50)	30	4140 = (30 x 138)
GoogLeNet				138	50 ≈ (6902/138)	30	1500 = (30 x 50)

Glossary: Observations (OBS), Test Set (TS), Validation Set (VS), Mini Batch Size (MBS), Iteration Per Epoch (IPE), Maximum Iterations (MI).

Consequently, the decision was made to extend the epoch count to 30 for all three models, given the necessity to examine the behavior of AlexNet, ResNet50, and GoogLeNet across a broader range of iterations. This adjustment aligns with a more coherent approach to studying CNNs for classification purposes. Additionally, the validation frequency was set to match the IPE, thereby conducting a validation at the end of each epoch. This strategy aims to be more conservative in computational cost, consequently reducing training time.

Upon completion of the training phase, algorithms were developed to generate Grad-CAM diagrams for each architecture by loading the trained models netALEXNET, netRESNET50, and netGOOGLENET (refer to software availability). The Grad-CAM analysis for multi-class classification of the categories Sus_G, Sus_R, and Non_D was conducted for each model.

## Results

**Figure 5.**
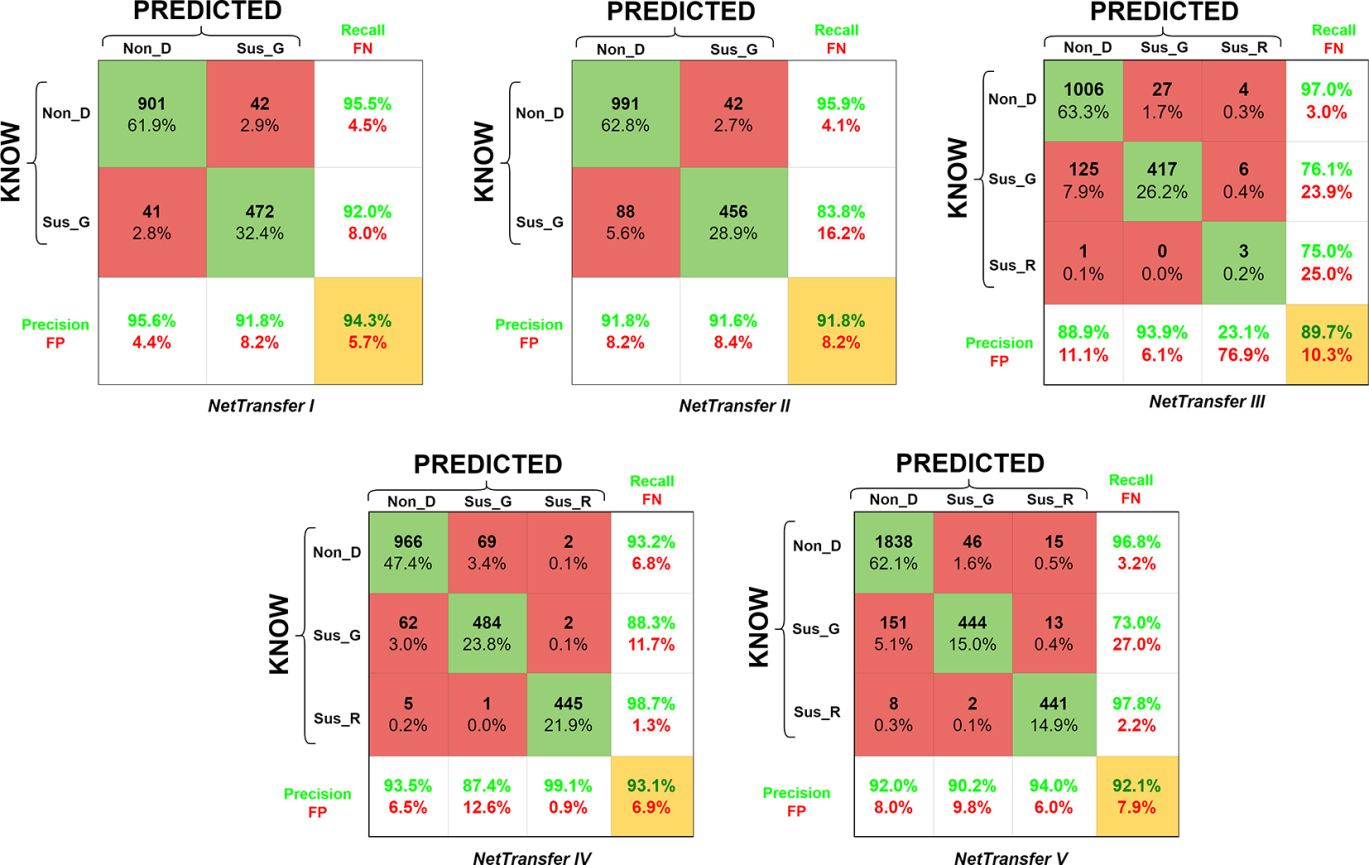
Confusion matrix for accuracy of the retrained AlexNet convolutional neural network on all datasets for the eye disease detection. Glossary: False Negative (FN), False Positive (FP), Non-disease (Non_D), glaucoma (Sus_G) and diabetic retinopathy (Sus_R).

The outcomes achieved through the application of transfer learning technique culminated in the development of five retrained AlexNet networks, hereafter referred to as NetTransfer networks. The confusion matrices for these NetTransfer networks are depicted in Figure 5, encompassing precision, recall, false positive (FP), false negative (FN), and accuracy values (highlighted in a yellow box). Furthermore, the matrices are structured such that the rows represent known values, while the columns indicate predicted values.

NetTransfer I network was only based on glaucoma and non-disease image cases existing in the LAG-database ( Table 2), training with these datasets lead to values of validation accuracy of 94.3%. Besides that, Non_D detection also presented values of 95.5% for recall (4.5% for FN), and values of 95.6% for the precision of the system (4.4% for FP).

NetTransfer II network was based on glaucoma and non-disease images cases existing in the LAG-database and the sjchoi86-HRF database ( Table 2), training with these datasets lead to values of validation accuracy of 91.8%. Besides that, Non_D detection presented values of 95.9% for recall (4.1% for FN), and values of 91.8% for the precision of the system (8.2% for FP).

NetTransfer III network was based on glaucoma, diabetic retinopathy and non-disease images cases existing in the LAG-database, sjchoi86-HRF database and the HRF database ( Table 2), training with these datasets lead to values of validation accuracy of 89.7%. Besides that, Non_D detection presented values of 97.0% for recall (3.0% for FN), and values of 88.9% for the precision of the system (11.1% for FP).

NetTarnsfer IV network was based on glaucoma, diabetic retinopathy and non-disease images cases existing in the LAG-database, sjchoi86-HRF database, HRF database and the APTOS database ( Table 2), training with these datasets lead to values of validation accuracy of 93.1%. Besides that, Non_D detection presented values of 93.2% for recall (6.8% for FN), and values of 93.5% for the precision of the system (6.5% for FP).

NetTransfer V network was based on glaucoma, diabetic retinopathy and non-disease images cases existing in the LAG-database, sjchoi86-HRF database, HRF database, APTOS database and ODIR database ( Table 2), training with these datasets lead to values of validation accuracy of 92.1%. Besides that, Non_D detection presented values of 96.8% for recall (3.2% for FN), and values of 92.0% for the precision of the system (8.0% for FP).

**Figure 6.**
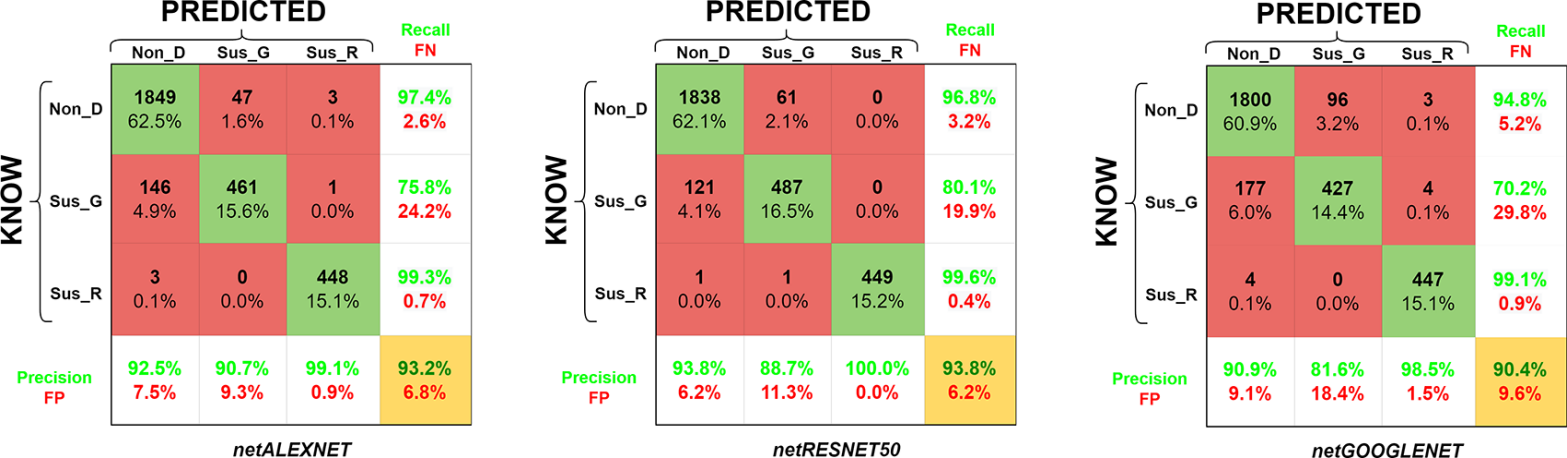
Confusion matrix for accuracy of the retrained AlexNet, ResNet50 & GoogLeNet convolutional neural networks for the eye disease detection. Glossary: False Negative (FN), False Positive (FP), Non-disease (Non_D), glaucoma (Sus_G) and diabetic retinopathy (Sus_R).

Similarly, the study includes an analysis of the transfer learning performance of the AlexNet network in comparison with other significant architectures, specifically ResNet50 and GoogLeNet. The models to which transfer learning was applied have been designated as netAlexNet, netResNet, and netGoogLeNet (see Figure 6).

**Figure 7.**
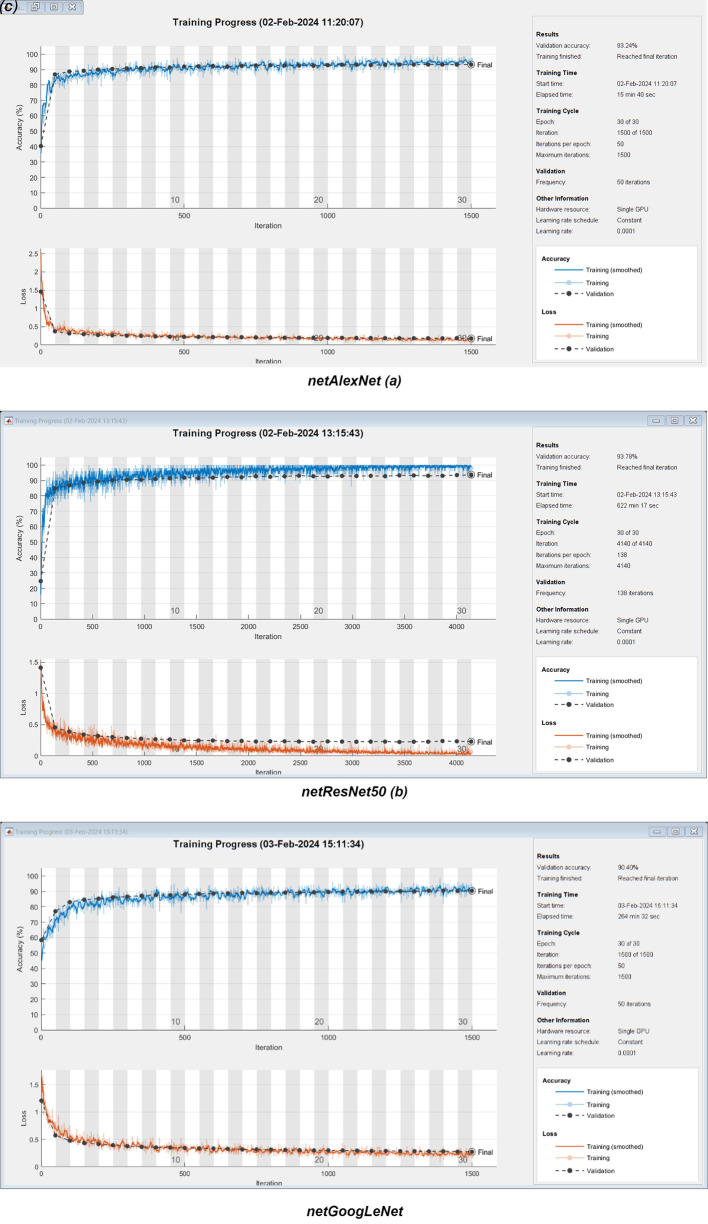
Performance Evolution and Training Metrics of netAlexNet, netResNet50, and GoogleNet Models.

The netAlexNet network was based on glaucoma, diabetic retinopathy, and non-diseased images from the LAG-database, sjchoi86-HRF database, HRF database, APTOS database, and ODIR database ( Table 4). Training with these observations returned a validation accuracy of 93.24%. Furthermore, detection of Non_D cases achieved a recall rate of 97.4% (2.6% for FN) and a precision of 92.5% (7.5% for FP). ResNet50 achieved a maximum validation accuracy of 93.8%, with recall and precision for Non_D detection at 96.8% and 93.8%, respectively. GoogleNet architecture attained a maximum validation accuracy of 90.4%, with its Non_D detection showing recall and precision rates of 94.8% and 90.9%, respectively. Additionally, training graphs for netAlexNet, netResNet50 network model are provided for enhanced understanding in a live-script (refer to software availability). These graphs detail the performance evolution during training and validation phases, alongside the training configurations and the duration of model training (see to Figure 7).

**Figure 8.**
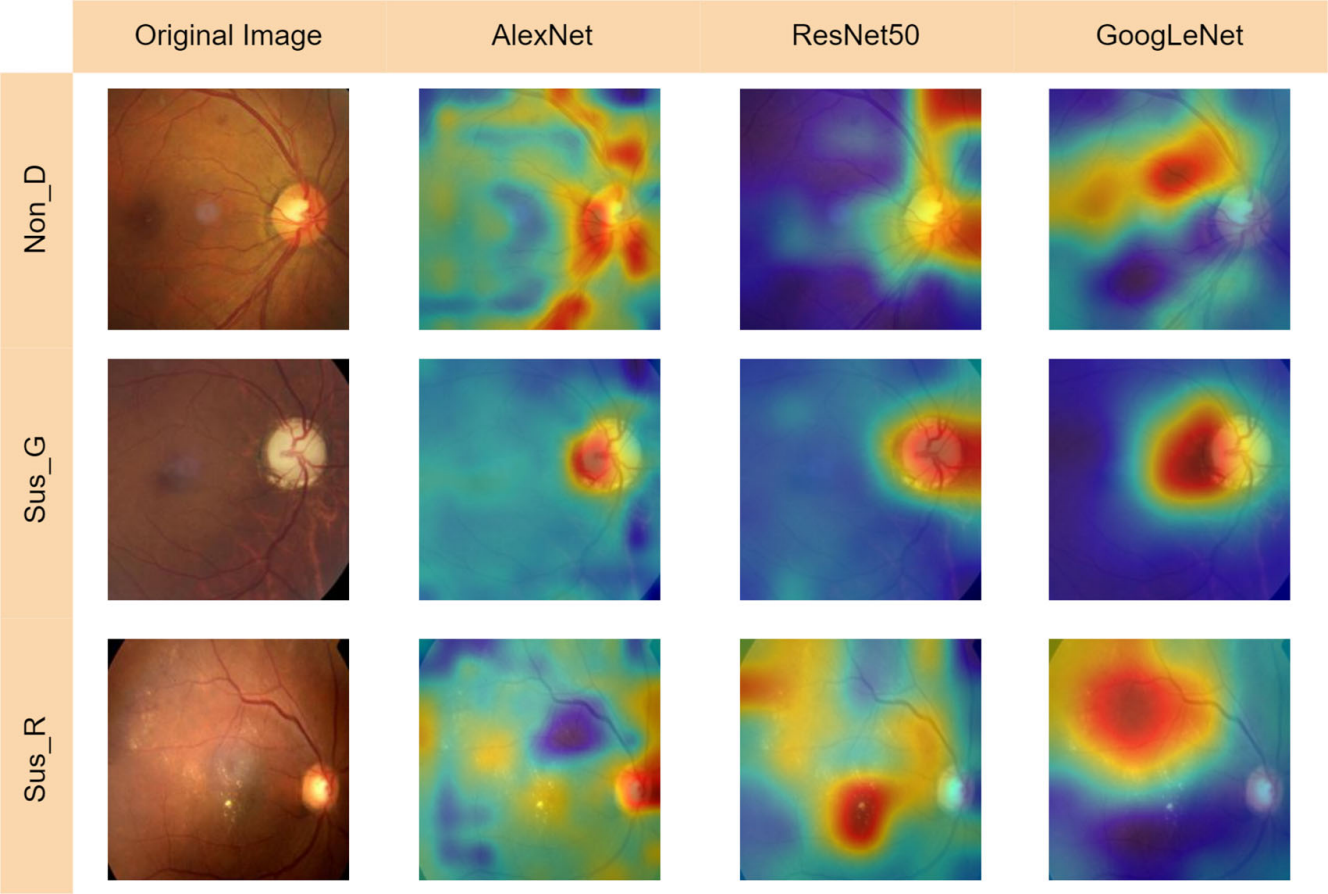
Grad-Cam for AlexNet, ResNet50 & GoogLeNet convolutional neural networks for the eye disease detection. Glossary: Non-disease (Non_D), glaucoma (Sus_G) and diabetic retinopathy (Sus_R).

Training duration for the netAlexNet network was observed at 15 minutes and 40 seconds, netResNet50 at 622 minutes and 17 seconds, and netGoogLeNet at 264 minutes and 32 seconds. Parameters such as EPOCHS, IPE, Validation Frequency, and Learning Rate are described for each model. Employing the netAlexNet model, in conjunction with the netResNet50 and netGoogLeNet neural networks, nine Grad-CAM heatmaps were generated to illuminate subtle differences in feature prioritization across the networks. This Grad-CAM involved a multi-class register across evaluated eye conditions, including Non_D, Sus_G, and Sus_R (see Figure 8).

## Discussion

Several works were presented for glaucoma detection using fundus photographs by calculating cup-disk-ratio (CDR). For example, Carrillo and coworkers ^41^ developed an autonomic detection method and a novel method for cup segmentation with a percentage of success of 88.5%. Another work from Anum Abdul and peers, ^42^ an algorithm was provided to detect CDR and hybrid textural and intensity features. Those features were used to classify the autonomous system, and it gave improvements in the results from previous studies that only used CDR, thanks to their hybrid approach, they reached an accuracy of 92%. Although the CDR characteristic was not utilized, the AlexNet methodology demonstrates comparable accuracy levels with NetTransfer V (92.1%) and netAlexNet (93.2%), matching the performance of previously cited methods without requiring CDR calculation.

In other more rigorous studies such as Xiangyu Chen work, ^43^ a deep CNN was developed with a total of six layers: two fully connected layers and four convolutional layers. The results drop scores of prediction from 71% to 83% from real images. On the other hand, Hanruo Liu and peers ^23^ made a deep learning system using a total of 241,032 images from 68,013 patients. In this work, every image was subjected to a multiple layers of grading system, in which graders were from students to senior specialists on glaucoma, from these they obtained good levels of sensitivity and specificity (82.20% and 70.40%). Compared to other CNN systems, the AlexNet-based detection systems (NetTransfer V & netAlexNet) exhibited results that are comparable and, in some cases, arguably superior in terms of accuracy, sensitivity, and specificity for detecting glaucoma and diabetic retinopathy.

Another related work from Almeida and peers, ^44^ uses image processing in MATLAB to improve the accuracy of glaucoma tests by extracting the most pertinent qualities of the images obtaining promising results with an accuracy, specificity, and sensitivity greater than 90%, which indicates that it gives an excellent start for us to assess the glaucoma diagnosis through AI. Although the system developed by Almeida and colleagues appears more specialized for glaucoma detection, NetTransfer V and netAlexNet offers the added benefit of simultaneously detecting multiple pathologies, including DR, which was another condition integrated into the detection framework.

Another study demonstrating superior results in glaucoma detection is that of Shinde, ^45^ where the application of two distinct architectures enabled perfect prediction (100%) of their images. However, as noted earlier, the versatility of the AlexNet network provides a comparative advantage by facilitating the differentiation and classification of multiple diseases. In contrast, a monolithic classification system may identify the absence of one disease (glaucoma), but overlook the presence of other pathologies, such as DR.

NetTransfer V & netAlexNet are also able to detect DR, it is also pertinent to compare it to other deep learning systems that were developed for DR detection. In the study realized by Rishab Gargeya and Theodore Leng, ^18^ they developed and evaluated a data-driven deep learning algorithm as a novel diagnostic tool for automated DR detection, which proved to reach high efficacy computer-aided model, with low-cost, which lead to correct DR diagnostics without depending on clinicians to examine and grade images manually.

A different study made by Shanthi and Sabeenian, ^19^ used a modified AlexNet CNN system for the detection of DR in a big data training of the network. Additionally, Amnia Salma and peers ^17^ develop a similar system, but they used GoogLeNet instead of AlexNet. While all of these systems follow similar principles to the NetTransfer V & netAlexNet propose systems, it is important to remark that the pre-trained networks acquired higher accuracies, sensitivities and specificities than the previously mentioned systems, mostly due to using a higher number datasets. The decision was made to extend the application of AlexNet for simultaneous classification of multiple diseases. The subsequent table provides a summary and comparison of the detection capabilities of the AlexNet pathology detection systems (NetTransfer V & netAlexNet) with those of all previously mentioned research, in addition to other significant studies not previously discussed ( Table 5).

**Table 5.** Comparison between related studies.

Detectable pathology	Detection method	Dataset (Size)	Type of channels	Number of classes	Performance measure				Ref.
					Accuracy (%)	Sensitivity (%)	Specificity (%)	AUC	
Glaucoma and diabetic retinopathy **( *NetTransferV*)**	CNN	LAG, APTOS, HRF, and ODIR (9860)	RGB	3	92.06	73.0 (Glaucoma) 97.80 (Retinopathy)	97.93 (Glaucoma) 98.78 (Retinopathy)	-	-
Glaucoma and diabetic retinopathy **(netAlexNet)**	CNN	LAG, APTOS, HRF, and ODIR (9860)	RGB	3	93.24	75.80 (Glaucoma) 99.30 (Retinopathy)	97.99 (Glaucoma) 99.83 (Retinopathy)		
Glaucoma	Algorithm to improve glaucoma detection using cup segmentation	Set of fundus images from the CPAG in Bucaramanga, Colombia	Gray scale, each RGB color channel independently	2	88.50	-	-	-	^41^
Glaucoma	Algorithm to detect glaucoma using a fusion of CDR and hybrid textural and intensity features	Local database of 50 fundus images with 15 glaucoma and 35 healthy images	Binary image, Green and RGB	2	92.00	100.00	88.00	-	^42^
Glaucoma	DCNN	ORIGA (650) and SCES (1676)	RGB	2	-	-	-	0.83 (ORIGA) and 0.88 (SCES)	^43^
Glaucoma	CNN	CGSA (241032)	Gray scale and RGB	3	-	82.20	70.40	0.82	^23^
Glaucoma	Direct Feed Neural Network	ACRIMA (705)	Green, gray scale and binary image	2	94.61	94.57	95.00	-	^44^
Glaucoma	CNN (LeNet & U-net)	IM-ONE, DRISHTI-GS, DRIONS-DB, JSIEC, NIO and DRIV	RGB	2	98.8 (ROI extraction) 100 (classification)	100	100	-	^45^
Diabetic retinopathy	Customized DCNN	EyePacs, MESSIDOR 2 and E-Ophtha (for testing) (75137)	RGB	2	-	74.00-94.00	80.00-98.00	0.94-0.97	^18^
Diabetic retinopathy	CNN (modified AlexNet)	Messidor (1190)	Green and RGB	4	95.60-96.60	88.00-96.00	97.30-96.60	-	^19^
Diabetic retinopathy	GoogleNet	Kaggle (200)	RGB	5	88.00	75.00	52.00	-	^17^
Diabetic retinopathy	Fused CNN512, CNN299, and CNN (YOLOv3, EfficientNetB0)	DDR (13673), and Kaggle (3662)	RGB	5	89.00	89.00	97.30	0.97	^16^
Diabetic retinopathy	ResNet (50 and 101) and VggNet-16	1607	RGB	2	ResNet-101 (98.88) ResNet-50 (93.00) VggNet-16 (71.39)	ResNet-101 (97.14)	ResNet-101 (97.65)	ResNet-101 (0.98)	^46^

In the benchmarking of AlexNet against other recognized architectures, specifically ResNet50 and GoogLeNet, the confusion matrices affirm that the netResNet50 model exhibits superior overall performance compared to the three models (refer to Figure 5). This superiority is partly attributed to flawless performance in the Sus_R category and enhanced performance in the Non_D category. The netResNet50 model is closely followed by its counterpart netAlexNet and, to a lesser extent, by GoogLeNet. However, the presentation of the training process through performance/validation graphs indicates a shorter training duration for netAlexNet in comparison to netResNet50 and netGoogLeNet ( Figure 6). This is largely attributed to the fewer number of layers in AlexNet, as opposed to GoogLeNet and ResNet50, the latter of which even necessitated a reduction in the MBS at the expense of increasing the IPE. Furthermore, within the performance graphs record, netResNet50 exhibited signs of overfitting, as evidenced by superior training performance over validation in consecutive epochs. Meanwhile, netGoogLeNet demonstrated performance similar to netAlexNet in the performance/validation process, which, despite a lower accuracy, did not show signs of overfitting.

The second row of images offers a detailed visualization of Grad-CAM outputs for the three networks applied to glaucomatous retinal images (see Figure 7). Initial examination of these images highlights a shared emphasis on the optic disk (OD) and optic nerve by all networks. However, a deeper analysis reveals significant differences in the degree and specificity of feature focus. ResNet50, recognized for its extensive reach, displays an extensive region of interest that goes beyond the OD boundaries, covering adjacent retinal areas. While this wide-ranging observation might seem beneficial, it adds complexity by including features outside the OD, which could complicate the classification task. On the other hand, GoogLeNet, known for its broad and encompassing analysis, indicates the widest area of interest, covering the entire OD and optic cup (OC), as well as the surrounding periphery. This extensive observation potentially facilitates a thorough evaluation of areas prone to pathological changes. However, its challenge lies in the indiscriminate encompassment of adjacent areas, possibly including anomalies not related to the disease under investigation, thus affecting diagnostic precision. Conversely, Grad-CAM outputs from the netAlexNet model, despite focusing on a more confined area, concentrate on crucial aspects such as the OD-OC ratio.

In the examination of DR images depicted in the third row of images (see Figure 8), a noticeable shift from the OD-focused analysis seen in glaucoma heatmaps is apparent. The GoogLeNet heatmap highlights a significant focus in the upper regions of the eye, likely due to increased vascularity. However, this focus might overlook potential manifestations of the disease in the lower regions and the OD, leading to possible misrecognition, especially in cases where DR pathology is primarily present in these areas. In contrast, the heatmap from ResNet50, while covering a wider area, demonstrates a less precise focus, capturing various ocular regions. This broad coverage aims to identify a wide range of retinal blood vessels and neurons but may result in a compromise between breadth and specificity, potentially affecting discernment capabilities.

Examination of eyes without disease reveals a focus on regions around ocular blood vessels and the OD in all three Grad-CAMs. The networks of AlexNet and ResNet50 extensively outline these areas. In contrast, GoogLeNet markedly neglects the OD, crucial for diagnosing both glaucoma and diabetic retinopathy, potentially leading to false negatives by missing clinically significant features, thus impacting recognition precision. This variation highlights the necessity for thorough feature assessment, particularly when minor anomalies are diagnostically critical.

Additionally, for the implementation of the AlexNet architecture on open-source language, the use of TensorFlow is endorse as a free open-source self-learning platform based on the Python language, mainly developed by Google. ^47^ Among its many available, Keras is identified as deep learning application programming interface (API) developed for Python and built on TensorFlow, where a user can build the proposed AlexNet equivalent model. The recommended model is the sequential model of Keras which allows a user to define the model as a series of convolutional layers with max pooling. ^48^

## Conclusions

In the presented research, the training of a CNN through the use of MATLAB software and its AlexNet tool, allowed the effective recognition of two eye diseases (glaucoma and DR) through retinal fundus images. Additionally, the use of open access databases allows the replicability and reproducibility of the present study. Being the APTOS, HRF and sjchoi86-HRF databases of immediate access. Meanwhile, LAG and ODIR are databases with access upon request. The implementation of the different databases (LAG, APTOS, HRF, ODIR, sjchoi86-HRF), proved to be effective in improving the prediction percentages of the different neural network trainings.

In general, the most common eye affections are presented through a series of symptoms, such as blurred vision, spots, glare, eye fatigue, dry eyes, among others. In this way, glaucoma proves to be a condition that damages the optic nerve and generally does not present any symptoms, until the person suffering from it perceives a decrease in vision in the final stages of the disease. Based on the foregoing, it is necessary to create tools that allow an effective detection of this type of affectation, for example CNN systems as an alternative, highly reliable in the automation of processes. Similarly, the study does not replace state-of-the-art technologies in the recognition of retinal pathology, nor to compete with identification systems that represent a new paradigm in the recording and analysis of retinal fundus images. Instead, offer an initial approach to enthusiasts interested in accessible recognition techniques through CNN models. Additionally, the research expanded its detection objective by incorporating a benchmark of models, complemented by a Grad-CAM analysis, through multi-class classification on the categories Sus_G, Sus_R and Non_D.

Future improvements to this algorithm could include the creation of a more user-friendly graphical interface for users who are not experts in programming language. In this way, the detection tasks will be based on the selection of options and not on the coding of algorithms. On the other hand, as previously mentioned, it is possible to replicate the AlexNet-CNN using Python, by using existing tools such as TensorFlow and Keras API. Therefore, a subsequent study will concentrate efforts on implementing the recognition system in the open-source language, to endorse the use of non-proprietary software in order to increase reproducibility.

### Software availability

MATLAB codes and scripts related to image processing, pre-processing & Training versions of the AlexNet Convolutional Neural Network ( *NetTransfers I-V*).

Source code available from: https://github.com/IscArias/EyeEvaluationSourceCode

Archived source code as at time of publication: https://doi.org/10.5281/zenodo.7098879 ^49^

License: 2-Clause BSD License

MATLAB codes and live scripts related to benchmarking of models & Grad-CAM (netAlexNET, netResNet50 & netGoogLeNet)

Archived source code as at time of publication: https://doi.org/10.5281/zenodo.10826326. ^50^

License: 2-Clause BSD License

## Data Availability

Free access - databases:
• Asia Pacific Tele-Ophthalmology Society (APTOS). The data download process is free and does not require authorization or registration. Follow the directions of the data host to reference the database. Link: https://www.kaggle.com/c7934597/resized-2015-2019-diabetic-retinopathy-detection/metadata/
•
High-Resolution Fundus (HRF) Image Database. The data download process is free and does not require authorization or registration. Follow the directions of the data host to reference the database. Link: https://www5.cs.fau.de/research/data/fundus-images/
• Sungjoon Choi High-Resolution Fundus (sjchoi86-HRF). The data download process is free and does not require authorization or registration. Follow the directions of the data host to reference the database. Link: https://github.com/cvblab/retina_dataset Access upon request – databases:
•
Large-scale attention based glaucoma (LAG). Database for academic purposes. An email must be written to the hosts who will provide a password to authorize the download. Once the key is obtained and entered, the download is free. It is not necessary to register on any additional platform. Follow the directions of the data host to reference the database. Link: https://github.com/smilell/AG-CNN

• Ocular Disease Intelligent Recognition (ODIR). Database for academic purposes. It is required to create an account and register on the platform, providing data on the user's institution, department and country. Once registered, a request must be submitted in order to download the database. Authorized access, the user can download the database freely. Follow the directions of the data host to reference the database. Link: https://odir2019.grand-challenge.org/dataset/ Additional Codes for Image Evaluation, Image Selection from Data Bases and Confusion Matrix Plotting. Source code available from: https://github.com/IscArias/EyeEvaluationSourceCode_Extra Archived source code as at time of publication: https://doi.org/10.5281/zenodo.7102618 ^51^ License: 2-Clause BSD License
