# Supplementary material for: Artificial intelligence based glaucoma and diabetic retinopathy detection using MATLAB — retrained AlexNet convolutional neural network
Source: F1000Res. 2024 Apr 3;12:14. Originally published 2023 Jan 5. [Version 2] doi: 10.12688/f1000research.122288.2 (PMC11143403; doi:10.12688/f1000research.122288.2)
Supplement: Peer review history

# Reviewer report: Reviewer response for version 2

**DOI:** 10.5256/f1000research.163934.r262369

**Published:** 2024-05-31

**Authors:** Tae Keun Yoo

**Affiliations:** 1 Ophthalmology, B&VIIT Eye Center, Seoul, South Korea

The authors well addressed my concerns.

 Since there are more diseases in ophthalmology, such as macular degeneration and ERM, the method proposed by the authors needs to be expanded to more classes in the future.

 In the future, it is expected that additional learning about various eye diseases will be possible with the advent of generative artificial intelligence.

Is the work clearly and accurately presented and does it cite the current literature?

Partly

If applicable, is the statistical analysis and its interpretation appropriate?

Partly

Are all the source data underlying the results available to ensure full reproducibility?

Yes

Is the study design appropriate and is the work technically sound?

Yes

Are the conclusions drawn adequately supported by the results?

Partly

Are sufficient details of methods and analysis provided to allow replication by others?

Yes

Reviewer Expertise:

Ophthalmology; deep learning; machine learning; fundus photography

I confirm that I have read this submission and believe that I have an appropriate level of expertise to confirm that it is of an acceptable scientific standard.

# Reviewer report: Reviewer response for version 2

**DOI:** 10.5256/f1000research.163934.r262370

**Published:** 2024-04-05

**Authors:** Creed Jones

**Affiliations:** 1 Electrical and Computer Engineering, Virginia Tech, Blacksburg, VA, USA

The revisions to the article are helpful. They improve the content without significantly changing the approach or results. My comments have been responded to.

Is the work clearly and accurately presented and does it cite the current literature?

Yes

If applicable, is the statistical analysis and its interpretation appropriate?

Yes

Are all the source data underlying the results available to ensure full reproducibility?

Yes

Is the study design appropriate and is the work technically sound?

Partly

Are the conclusions drawn adequately supported by the results?

Yes

Are sufficient details of methods and analysis provided to allow replication by others?

Partly

Reviewer Expertise:

machine learning, computer vision, eye diseases, biometric identification.

I confirm that I have read this submission and believe that I have an appropriate level of expertise to confirm that it is of an acceptable scientific standard.

# Reviewer report: Reviewer response for version 1

**DOI:** 10.5256/f1000research.134260.r213588

**Published:** 2023-10-27

**Authors:** Tae Keun Yoo

**Affiliations:** 1 Ophthalmology, B&VIIT Eye Center, Seoul, South Korea

The authors built a CNN model to classify glaucoma, diabetic retinopathy, and controls in the fundus photography domain using MATLAB.

1. Alexnet is the first CNN model known in the world and the most primitive structure. It is one of the oldest architecture of CNNs. Why did the authors choose alexnet in defiance of many recent advances in CNN? At least use and compare resnet. It is all provided by Matlab.
2. Matlab provides a different deep learning toolbox for each version. Write in detail which version and which toolbox the authors used.
3. Provide Grad-CAM heatmaps.
4. Please describe the partitioning process for the training and test sets in detail.
5. Considering recent advances of deep learning in ophthalmology domains, the contribution of this study is too limited. Please review recent advances and show the strengths of this study.
6. Many important papers in this fields are omitted in the manuscript. Please discuss several siginficant previous works and important products of fundus photography reading devices for diabetic retinopathy.

Is the work clearly and accurately presented and does it cite the current literature?

Partly

If applicable, is the statistical analysis and its interpretation appropriate?

Partly

Are all the source data underlying the results available to ensure full reproducibility?

Yes

Is the study design appropriate and is the work technically sound?

Yes

Are the conclusions drawn adequately supported by the results?

Partly

Are sufficient details of methods and analysis provided to allow replication by others?

Yes

Reviewer Expertise:

Ophthalmology; deep learning; machine learning; fundus photography

I confirm that I have read this submission and believe that I have an appropriate level of expertise to confirm that it is of an acceptable scientific standard, however I have significant reservations, as outlined above.

# Response

**Published:** 2024-03-17

**Authors:** Angel Arias

**Affiliations:** Universidad de Investigación de Tecnología Experimental Yachay, Ecuador

**1. Alexnet is the first CNN model known in the world and the most primitive structure. It is one of the oldest architecture of CNNs. Why did the authors choose alexnet in defiance of many recent advances in CNN? At least use and compare resnet. It is all provided by Matlab.**

 AlexNet has remained a significant neural network over time, owing to the simplicity of its architecture, which allows operation without the need for extensive computational resources. The observation provided led to the realization that benchmarking models was essential to better understand the performance of the study relative to other significant architectures. Consequently, comparisons were not only made with the ResNet50 architecture but also included GoogLeNet, thereby enriching the study with a broader variety of architectural analyses.

**2. Matlab provides a different deep learning toolbox for each version. Write in detail which version and which toolbox the authors used.**

 This observation was fully addressed by adding a new section titled "Operational Resources." This section not only describes the software used, including the version of MATLAB employed for the study, but also details the hardware utilized. Appreciation is expressed for highlighting this theoretical component, which was an oversight not previously considered in the study.

**3. Provide Grad-CAM heatmaps.**

 Given the incorporation of model benchmarking in the updated version, the opportunity was taken to conduct a Grad-CAM analysis using the three trained models: netAlexNet, netResNet50, and netGoogLeNet. The Grad-CAM analysis was performed for each model across the three identification conditions considered: non-disease, suspicious glaucoma, and suspicious diabetic retinopathy. Appreciation is extended for the request of this addition, as it enhances the comprehensiveness of the work through the interpretative insight provided by the Grad-CAM heatmaps for each model and evaluated condition.

**4. Please describe the partitioning process for the training and test sets in detail.**

 The image processing section has been enhanced to address previous ambiguities and provide a more comprehensive understanding. Furthermore, a figure illustrating the data partitioning volume for segregating images into validation and training datasets has been added. A mathematical framework for model training parameters, contingent on the number of observations in the training and validation sets, has been established. This framework includes detailed descriptions of Mini Batch Size, Iterations Per Epoch, Training Set (TS), and Validation Set (VS), thereby rendering the information of the study to be more relevant and concise through the requested corrections.

**5.Considering recent advances of deep learning in ophthalmology domains, the contribution of this study is too limited. Please review recent advances and show the strengths of this study.**

 The development of predictive systems in the field of ophthalmology has been on the rise for several years with the use of Convolutional Neural Networks (CNNs) and other types of systems, many of which emulate the structures of existing networks for prediction purposes. Within this context, the presented work does not signify a paradigm shift in the state of the art, as it aligns with concepts previously implemented. Nonetheless, the study demonstrates significant utility and importance, highlighting that transfer learning via AlexNet yields highly promising results, potentially surpassing some detection methods, networks built from scratch, or studies that employed pre-established network structures. This approach advocates for the adoption of transfer learning and a streamlined methodology that does not require complex data pre-processing, thereby simplifying classification tasks and aligning with existing predictive systems.

**6. Many important papers in this fields are omitted in the manuscript. Please discuss several significant previous works and important products of fundus photography reading devices for diabetic retinopathy.**

 In response to this observation, a select number of contemporary and pertinent studies within the field, related to predictive systems for these pathologies, have been incorporated. It is noted that the aim of this work is not to compete with or replace established systems, nor is it intended to be perceived as comparable to specialized retinal fundus image technologies. Rather, it is approached as an initial step towards the development of a system capable of ensuring efficient and accessible identification tasks, with considerations for future enhancements.

# Reviewer report: Reviewer response for version 1

**DOI:** 10.5256/f1000research.134260.r161036

**Published:** 2023-02-06

**Authors:** Creed Jones

**Affiliations:** 1 Electrical and Computer Engineering, Virginia Tech, Blacksburg, VA, USA

The article presents a CNN implementation using MATLAB to determine the presence of glaucoma using labeled retinal images. The result and its conclusions are very interesting, and the open and freely verifiable nature of the work is great.

 Detailed Comments -

1. The abstract should clarify the "two classes" and "three classes" referred to.
2. The authors refer to a "red color filter" that is added to grayscale images, which is not necessary if RGB images are available. This is very confusing and needs both justification and explanation.
3. The use of 6 epochs needs to be justified.
4. The presentation of the confusion matrices is very clear and good.
5. It's not clear whether external knowledge of diabetes diagnoses was used (or could be used) in distinguishing DR from glaucoma.
6. Clearly calling out the available databases is very helpful for replicating/extending the results.
7.  Grammar throughout is good but needs improvement. Look at use of "The" at the start of sentences. Also, is there a misspelling in the title of the confusion matrices; should "PREDICTEC" be "PREDICTED"?

Is the work clearly and accurately presented and does it cite the current literature?

Yes

If applicable, is the statistical analysis and its interpretation appropriate?

Yes

Are all the source data underlying the results available to ensure full reproducibility?

Yes

Is the study design appropriate and is the work technically sound?

Partly

Are the conclusions drawn adequately supported by the results?

Yes

Are sufficient details of methods and analysis provided to allow replication by others?

Partly

Reviewer Expertise:

machine learning, computer vision, eye diseases, biometric identification.

I confirm that I have read this submission and believe that I have an appropriate level of expertise to confirm that it is of an acceptable scientific standard, however I have significant reservations, as outlined above.

# Response

**Published:** 2024-03-17

**Authors:** Angel Arias

**Affiliations:** Universidad de Investigación de Tecnología Experimental Yachay, Ecuador

**1. The abstract should clarify the "two classes" and "three classes" referred to.The abstract should clarify the "two classes" and "three classes" referred to.**

 Thank you for this observation, as the omission was not previously recognized. To address this oversight, the abstract has been revised for greater precision and conciseness. This revision now explicitly references the classes classified during the study and includes information detailing the new implementations of the study.

**2. The authors refer to a "red color filter" that is added to grayscale images, which is not necessary if RGB images are available. This is very confusing and needs both justification and explanation.**

 The mention of the red color filter has been removed from the image and text pre-processing flowchart within the manuscript. This feature was initially presented as an optional additional functionality within our code for the convenience of the reader. However, it lacks utility in the context of this study, given that the databases utilized are in RGB format. Acknowledgment of this detail is appreciated, as it was a component without relevance to the scope of the study that was not addressed throughout its duration.

**3. The use of 6 epochs needs to be justified.**

 In transfer learning, the objective is to leverage features already learned by the pre-trained model. It was posited that fewer epochs were necessary, as the focus shifts towards "fine-tuning" the weights in the final layers of the model for a new specific task rather than learning these features from scratch. However, following the observation made, it was recognized that this aspect required improvement given it had been inadequately covered, this version includes elements like MiniBatchSize, iterations per epoch, and validation frequency for the theoretical background. Consequently, additional training sessions with AlexNet, ResNet50, and GoogLeNet were conducted for model benchmarking, with an increased number of epochs aligning with more traditional training cycles.

**4. The presentation of the confusion matrices is very clear and good.**

 The design of the matrices, as well as all figures within the study, are original creations. Furthermore, enhancements have been implemented to ensure the images are more comprehensible and visually clear.

**5. It's not clear whether external knowledge of diabetes diagnoses was used (or could be used) in distinguishing DR from glaucoma.**

 External knowledge from medical experts on glaucoma and diabetic retinopathy is leveraged for generating extensive, accurately labelled databases. This foundation enables the data to undergo analysis, providing a basis of truth for the development of classification models by non-medical professionals. Moreover, while physicians rely on physiological criteria and specific medical evaluations for diagnosing retinal diseases using images, Convolutional Neural Networks (CNNs) identify patterns within a set of images that may or may not correlate with characteristic features of retinal diseases. Consequently, the application of Grad-CAM becomes essential to ascertain the focal points of a trained model during the discriminative process of recognition. This observation is appreciated, as it has facilitated the enhancement of previously provided information in the article and the correction of potential inaccuracies.

**6. Clearly calling out the available databases is very helpful for replicating/extending the results.**

 The rationale for providing a detailed description and citation of the databases utilized was to ensure access to information for individuals interested in conducting related studies on retinal fundus images. Appreciation is extended for acknowledging the thoroughness of the descriptions provided.

**7. Grammar throughout is good but needs improvement. Look at use of "The" at the start of sentences. Also, is there a misspelling in the title of the confusion matrices; should "PREDICTEC" be "PREDICTED"?**

 Consequently, a decision was made to enhance the overall clarity of the text, including the revision of sections to ensure the narrative is presented in the third person. It is anticipated that these modifications will contribute to a more assertive tone throughout the document.
